# Extracellular matrix stiffness: mechanisms in tumor progression and therapeutic potential in cancer

**DOI:** 10.1186/s40164-025-00647-2

**Published:** 2025-04-10

**Authors:** Meiling Zhang, Bin Zhang

**Affiliations:** 1 School of Basic Medicine, China Three Gorges University, 8 Daxue Road, Yichang, 443002 Hubei China; 2 Central Laboratory, The First Affiliated Hospital of Jinan University, No. 613 Huangpu West Road, Tianhe District, Guangzhou, 510627 Guangdong China

**Keywords:** Tumor microenvironment, Extracellular matrix, Stiffness, Mechanical sensor, Cancer therapy

## Abstract

Tumor microenvironment (TME) is a complex ecosystem composed of both cellular and non-cellular components that surround tumor tissue. The extracellular matrix (ECM) is a key component of the TME, performing multiple essential functions by providing mechanical support, shaping the TME, regulating metabolism and signaling, and modulating immune responses, all of which profoundly influence cell behavior. The quantity and cross-linking status of stromal components are primary determinants of tissue stiffness. During tumor development, ECM stiffness not only serves as a barrier to hinder drug delivery but also promotes cancer progression by inducing mechanical stimulation that activates cell membrane receptors and mechanical sensors. Thus, a comprehensive understanding of how ECM stiffness regulates tumor progression is crucial for identifying potential therapeutic targets for cancer. This review examines the effects of ECM stiffness on tumor progression, encompassing proliferation, migration, metastasis, drug resistance, angiogenesis, epithelial-mesenchymal transition (EMT), immune evasion, stemness, metabolic reprogramming, and genomic stability. Finally, we explore therapeutic strategies that target ECM stiffness and their implications for tumor progression.

## Introduction

TME generally refers to the complex ecosystem around tumor tissues, which contains not only the tumor cells themselves, but also stromal cells (cancer-associated fibroblasts (CAFs), pericytes, immune cells, mesenchymal stromal cells (MSCs), endothelial cells (ECs)), the blood vessels and lymphatic system, ECM and other secreted molecules, such as growth factors, cytokines, hormones, chemokines, and extracellular vesicles [1]. Initially, these host cells were treated as bystanders of tumorigenes [2]. However, due to the results of mechanistic studies, including in preclinical tumor models, it has been found that TME determines tumor growth, invasion, metastasis, and treatment resistance through the interaction of cells and their secreted signaling molecules as well as with ECM, among which ECM is considered to play a key role in the pathogenesis of cancer [2].

ECM forms scaffolds for tissues and organs by generating supramolecular aggregates such as fibrils and lamellar networks. It is a complex network composed of fibrin (collagen, elastin), glycosaminoglycans (eg, hyaluronic acid [HA]), proteoglycans (chondroitin sulfate and heparin sulfate), and glycoproteins (fibronectin 1 [FN1], laminin, and tenasin C [TNC]). ECM is dynamically synthesized, secreted, assembled, and modified by a variety of cell types, among which CAFs are the main regulators of ECM composition by secreting collagen, elastin, laminin, proteoglycans, MMPs and LOX/LOXL2 [3]. Beyond its biochemical components, the ECM’s biophysical properties—morphology, stiffness, molecular density, and tension—are crucial. It not only impedes drug delivery but also imparts chemical and mechanical cues that influence cell shape, metabolism, function, migration, proliferation, and differentiation. In addition to biochemical components such as covalent cross-links between molecules, the biophysical characteristics of the ECM include its morphology, stiffness, molecular density, and tension.

The ECM not only serves as a barrier against drug penetration and immune cell entry, but also provides chemical signals and mechanical forces that affect cell morphology, proliferation, migration, proliferation, metabolism, and differentiation. Mechanical stimuli such as compression, matrix stiffness, and fluid dynamics convey positional and environmental information to cells, prompting responses that aid tissue repair and homeostasis until mechanical equilibrium is attained. Matrix stiffness, being a crucial biomechanical attribute, is characterized by the material’s opposition to deformation when subjected to a force applied at an extremely low rate (quasi-static). Quantitatively, this property is assessed using the elastic modulus [4]. Within a physiological framework, the stiffness of the ECM is delicately balanced and plays a crucial role in sustaining cellular equilibrium and tissue structure.

Nevertheless, abnormal alterations in ECM stiffness have been linked to numerous disease states, notably in cancer, where the ECM undergoes dynamic remodeling during tumor progression. These changes are reflected in its composition, spatial structure, fiber alignment orientation, and especially biomechanical properties, which jointly regulate ECM stiffness [5]. Elevated ECM stiffness activates cellular responses through mechanical signal transduction pathways, such as Integrin/FAK and YAP/TAZ signaling pathways [6], driving tumor evolution to a malignant phenotype [7, 8]. Cancerous tissues typically exhibit greater stiffness than normal or adjacent tissues. For example, breast cancer tissue (5–10 kPa) is stiffer than normal breast tissue (800 Pa) [9]. Normal hepatic tissue has a stiffness of less than 6 kPa, while values higher than 8–12 kPa are indicative of conditions such as fibrosis or cirrhosis that may result in the development of liver cancer (HCC) [10]. Normal healthy pancreas tissue has a stiffness of 1–3 kPa, whereas pancreatic carcinoma tissue has a stiffness of over 4 kPa [11, 12]. For lung tissue, it has been suggested that solid tumors of the lung are stiffer (20–30 kPa) than normal lung parenchyma (150–200 Pa) [13]. Glioblastomas (7–27 kPa) are stiffer than non-malignant gliosis (50–450 Pa) [14], similarly, in patients with gastric cancer (7kpa) than in normal gastric tissue (0.5–1kpa) [15]. The results indicated that the stiffness of the solid tumor was higher than that of the normal tissue.

As a key component of the TME, elevated ECM stiffness is a salient feature of cancer. Malignant cells induce elevated ECM stiffness during cancer development, which reciprocally influences cancer cell characteristics. When cancer cells communicate with the ECM, they activate signal transduction. Therefore, a comprehensive understanding of how ECM stiffness regulates cancer progression will help to identify potential therapeutic targets for cancer.

This review comprehensively examines factors influencing matrix stiffening, such as matrix deposition and collagen cross-linking, and explores the impacts of ECM stiffness on tumor proliferation, invasion, metastasis, drug resistance, angiogenesis, immune escape, EMT, stemness, metabolic reprogramming, and genomic stability. Subsequently, it discusses potential therapeutic targets for modulating ECM stiffness and tumor progression, followed by an exploration of challenges associated with targeting ECM-related signals in cancer therapy.

## Key factors for regulating ECM stiffness

Previous research has shown that in solid tumors, tissue sclerosis is regulated by both tumor cells and stromal cells in the TME. The increase in ECM stiffness in cancer sclerosis primarily results from the accumulation of ECM proteins, particularly due to collagen rearrangement. The activation of various crucial signaling pathways, such as Transforming Growth Factor β (TGF-β), Interleukin-33 (IL-33), insulin-like growth factor (IGF)/IGF1R, FAK/Src, RhoA/Rock, MAPK and phosphatidylinositol 3 kinase (PI3K)/Akt, promotes the production of ECM proteins. The lysyl oxidase (LOX) family and the procollagen-Lys, 2-oxoglutarate 5-dioxygenase (PLOD) family, particularly peptidyl-proline cis–trans isomerase (PPIase) and lysyl hydroxylase 2 (LH2), play essential roles in the collagen rearrangement process.

### Matrix deposition

**Fig. 1:**
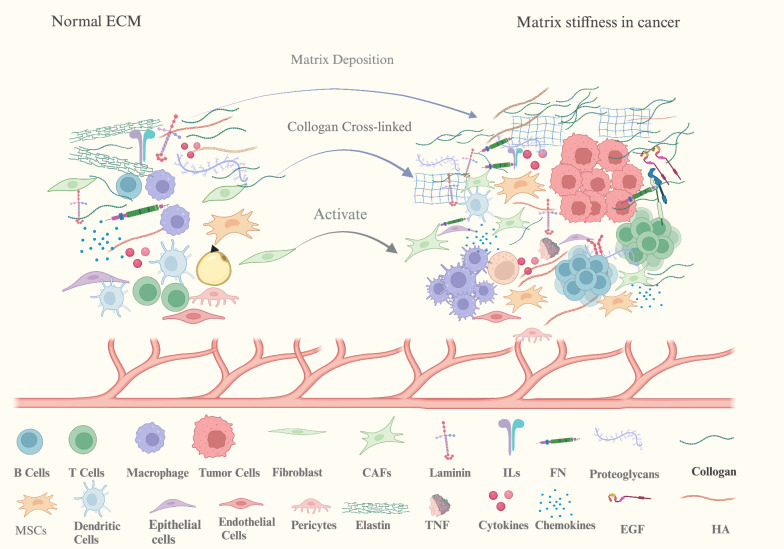
Schematic illustration of ECM components in normal tissue (left) and the TME (right). Matrix stiffness is mainly related to excessive collagen and HA. Both cytokines or TGFβ are involved in the ECM stiffness process, and LOX is involved in collagen crosslinking. ECM stiffness interacts with tumor cells and stromal cells, thus creating a vicious cycle

The interaction between CAF and ECM stiffness can promote matrix deposition, thereby enhancing ECM stiffness. Specifically, high matrix stiffness can activate fibroblasts, leading to the secretion of collagen, FN, GAGs, and other components, thus forming a positive cycle [16]. At the same time, matrix stiffness also induces the expression of palladin, collagen IV, and collagen VIα1 chains by promoting the overexpression of Twist1 in CAFs [17, 18]. Thus, matrix deposition is promoted. In addition, CAFs respond to the increase of matrix stiffness by reducing the expression and secretion of matrix metalloproteinase 9 (MMP-9) and upregulating the secretion of inhibitor of metalloproteinase 1 (TIMP-1), leading to tissue sclerosis caused by matrix accumulation [16]. In turn, the varying matrix stiffness enhances the contractile and secretory capacity of cardiac fibroblasts through piezoelectric receptors [19], thereby regulating the localization of transcription factors and affecting the phenotypic transformation of myofibroblasts and collagen production [19]. For example, during palatal healing, periostin increases ECM stiffness through FN synthesis and the integrin-β1/RhoA pathway, thereby affecting fibroblast differentiation and contraction [17–19]. Rho-associated protein kinase (ROCK) is an important mechanosensor between matrix stiffness and tumor cells [18]. [18] Rock controls the production of collagen, FN, and laminin through the β-catenin signaling pathway. In addition, ROCK2 upregulates ECM stiffness by inhibiting p21 expression and enhancing NF-κB and TNC expression. Furthermore, in HCC, matrix stiffness triggers P300 phosphorylation through activation of RhoA/AKT signaling pathway, leading to P300 nuclear translocation and increased gene transcription, which in turn activates hepatic stellate cells (Fig. 1).

YAP is required for CAFs to promote matrix sclerosis, acting as a marker of CAFs at early tumor stages and as an inducer of mechanical activation of CAF function [5]. Specifically, ECM stiffness and contraction act on YAP through activation of Src, which promotes its nuclear translocation function by directly phosphorylating YAP. Thus, the binding of YAP to TEAD is enhanced. Enhanced YAP function further leads to increased α-SMA expression, which not only enhances myofibroblast contractility but also maintains the CAF phenotype. In this state of high contraction, myofibroblasts can apply greater traction forces to the fibrotic ECM, resulting in additional matrix reorganization and increased stiffness, which enhances the potential activation of TGF-β and thereby completes the vicious cycle [19].

In addition to the mechanisms described above, hypoxia also induces increased ECM stiffness. For example, cancer cells and CAFs secrete IL-6 to induce the expression of hypoxia-inducible factor-1α (HIF-1α). HIF-1α increases the synthesis rate of ECM proteins and promotes the expression of several hydroxylases and LOX, which further promotes ECM stiffness [20].

Many growth factors can stimulate ECM stiffness in the TME. Cytokines such as IL-33, TGF-β, platelet-derived growth factor receptor α (PDGFRα), and tumor necrosis factor α (TNF-α) have been identified as factors that enhance the activation and proliferation of CAFs, thereby promoting the synthesis and deposition of matrix components. IL-33 enhances fibrosis through the activation of immune cells. Specifically, in the liver, IL-33 stimulates the proliferation of resident innate lymphocytes, leading to the production of IL-13, which in turn activates hepatic stellate cells. IL-13 contributes to fibrosis by inducing collagen accumulation, down-regulating MMPs, and attracting profibrotic innate immune cells. Meanwhile, IL-13 also elevates the expression of TGF-βand facilitates the transformation of fibroblasts into myofibroblasts [21].

TGF-β is normally secreted by fibroblasts and is one of the most potent stimulators of fibrosis, which enhances COL1A1, COL3A1, TIMP1, and about 60 other ECM-related genes directly by facilitating nuclear translocation of transcription factors SMAD2-SMAD3 complex. In the breast interstitium, activated PDGFRα causes increased HA and collagen deposition in breast fibroblasts, inducing fibrosis of the mammary fat pad, and it is observed that fibrosis of the mammary gland significantly increases the overall stiffness of the breast as measured by atomic force microscopy [22].

In addition to the growth factors mentioned above, in tumor cells, methylation of RASSF1A promoter leads to elevated expression of YAP1 and P4HA2, which together promote collagen deposition in the ECM. This process accelerates collagen deposition by promoting procollagen folding and processing through upregulation of the chaperone heat shock protein 47 (HSP47). HSP47 also interacts with matrix proteins such as decorin, lumican, and fibromodulin to promote the secretion of collagen into ECM [7]. In addition, the acidic and cysteine-rich proteome acts as another matrix chaperone that binds collagen to prevent its degradation and promotes proper collagen assembly. Finally, aging MSCs may also elevate collagen density and matrix stiffness. Conversely, tumor stiffness in turn modulates the differentiation of MSCs and the reprogramming of MSCs in order to enhance their tumor promoting activity.

### Collagen cross-linking

The density and arrangement of collagen and elastic fibers are crucial determinants of the stiffness of the ECM. Cross-linking of collagen and elastic fibers, along with the presence of highly ordered matrix fibers, significantly influences matrix stiffness [23]. Fibroblasts synthesize most ECM components and coordinate their assembly and spatial distribution. Collagen cross-linking is almost exclusively mediated by LOX [24], a copper-dependent, secreted amino oxidase encoded by the PLOD family. LOX hydroxylates Lys residues, resulting in increased Hyl aldehyde-derived collagen (HLCC) and decreased Lys aldehyde-derived collagen cross-linking (LCC), thereby altering matrix properties. HLCC is predominantly observed in tissues with high stiffness, such as bone, while LCC is more prevalent in tissues with lower stiffness, such as connective tissue. The LOX family facilitates the conversion of peptide residues lysine (Lys) and hydroxylysine (Hyl) into reactive aldehydes (Lysald and Hylald), which then combine with adjacent Lys, Hyl, and Histidine residues to form a variety of crosslinks, thereby enhancing ECM structural stiffness [25].

The LOX family comprises five copper-dependent amino oxidases: LOX and LOX-like (LOXL) proteins, including LOXL-1, LOXL-2, LOXL-3, and LOXL-4. These enzymes are found in a wide range of tissues and organs, such as aorta, heart, lung, and brain [26]. LOX secretion is regulated by multiple signaling molecules. For instance, tumor cells trigger a sequence of activities, such as the upregulation of integrin α7, the secretion of TGF-β1, the phosphorylation of focal adhesion kinase (FAK)/Src, and the activation of extracellular signal-regulated kinase (ERK) 1/2 [25], culminating in LOX secretion to catalyze collagen cross-linking and increase matrix stiffness [7]. Transcription factors such as Twist1 and ZEB1, which promote EMT and cancer metastasis, can increase the expression of LOX and LOXL2 by inhibiting miR-200, thereby enhancing collagen cross-linking and matrix stiffness. In HCC cells, higher matrix stiffness significantly upregulates the expression of LOXL2 by activating the integrin β1/α5/JNK/c-JUN signaling pathway [27].

**Fig. 2:**
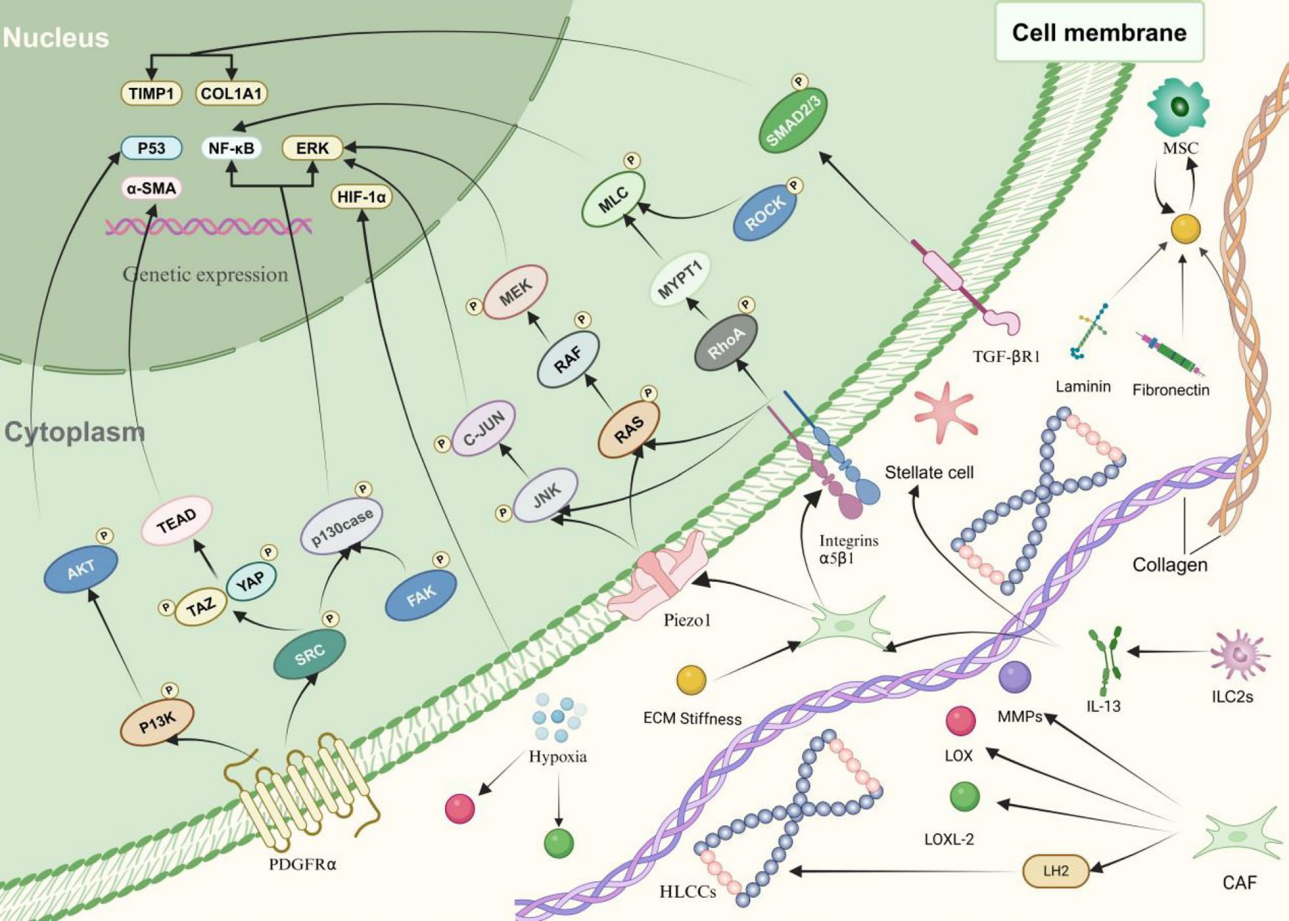
Key factors and signaling pathways inducing ECM stiffness. Stiffness activates receptors such as integrin, Piezo, and PDGFR, which sense mechanical signals from the ECM and activate downstream pathways, including FAK/SRC, ERK, AKT, β-catenin, RhoA-ROCK, and YAP/TAZ to induce ECM stiffness enhancement. Stromal cells such as CAF, senescent MSC and factors such as TGFβ and hypoxia play a major role in stiffness-mediated properties

LH2 specifically hydroxylates the telopeptide Lys residues of collagen, crucial for the formation of stable crosslinks. Both tumor cells and CAFs secrete LH2, which increases tumor stiffness by upregulating LOXL2 in response to high matrix stiffness. For example, in lung tissue, LH2 produced by lung cancer cells increases HLCCs and enhances lung cancer tissue stiffness [16]. Conversely, transcription factors HIF1-α, SMAD, and GATA3 contribute to hypoxia-induced matrix stiffness by directly inducing LH2 overexpression. Additionally, PPIase FKBP65, tissue glutaminase, and small G proteins, including Ras and Rho family members, play critical roles in collagen fiber properties and production. FKBP10 interacts with LH2 to promote its dimerization, thereby enhancing collagen pyridine cross-linking. Tissue glutaminase crosslinks collagen to sclerose pancreatic tumor tissue. Small G proteins alter collagen alignment to promote matrix stiffness [26]. Based on these findings, pharmacological inhibitors of these enzymes can be developed to reduce collagen cross-linking in tumors and inhibit metastasis in tumor-bearing animals (Fig. 2) [25].

## Effects of ECM stiffness on tumor progression

### Effect of ECM stiffness on tumors—proliferation

ECM stiffness in TME is a key factor in regulating the proliferation of tumors, β-catenin and Rho/ROCK signaling pathways play a central role in affecting cell behavior and tissue structure by regulating the cytoskeleton and signal transduction pathways. β-catenin plays a vital role in epidermal homeostasis and tumorigenesis. β-catenin is involved in the regulation of progenitor cell proliferation by regulating the transcription of Tcf/Lef target genes. In addition, as a core element of the transcriptional pathway in response to mechanical stimulation, β-catenin can sense changes in ECM stiffness, which in turn regulates the cytoskeleton and signaling pathways, affecting cell proliferation and tumor progression.

The Rho/ROCK pathway is a central mediator in the cellular response to changes in ECM stiffness, enabling cells to adjust to variations in external forces and maintaining the stability of tissue structures by enhancing actin-myosin-mediated cellular tension. Experimental models have shown that conditionally activated ROCK2 can increase tissue stiffness by increasing collagen, which further activates nuclear transcriptional activity by stabilizing β-catenin [28], leading to excessive cell proliferation and tissue thickening [28]. ECM stiffness directly regulates the proliferation ability of HCC and other tumor types. In the ECM with higher stiffness, tumor cells such as Huh7 and HepG2 showed significant proliferation, mainly through the activation of ERK, protein kinase B (Akt), and signal transducer and activator of transcription 3 (STAT3) signaling pathways [29]. In addition, β1-integrin and FAK are also involved in the stiffness-dependent regulation of HCC cell proliferation [30]. Finally, although higher matrix stiffness did not alter YAP expression levels, it led to decreased phosphorylated YAP, facilitated its nuclear localization [31], and influenced gene expression [32].

**Fig. 3:**
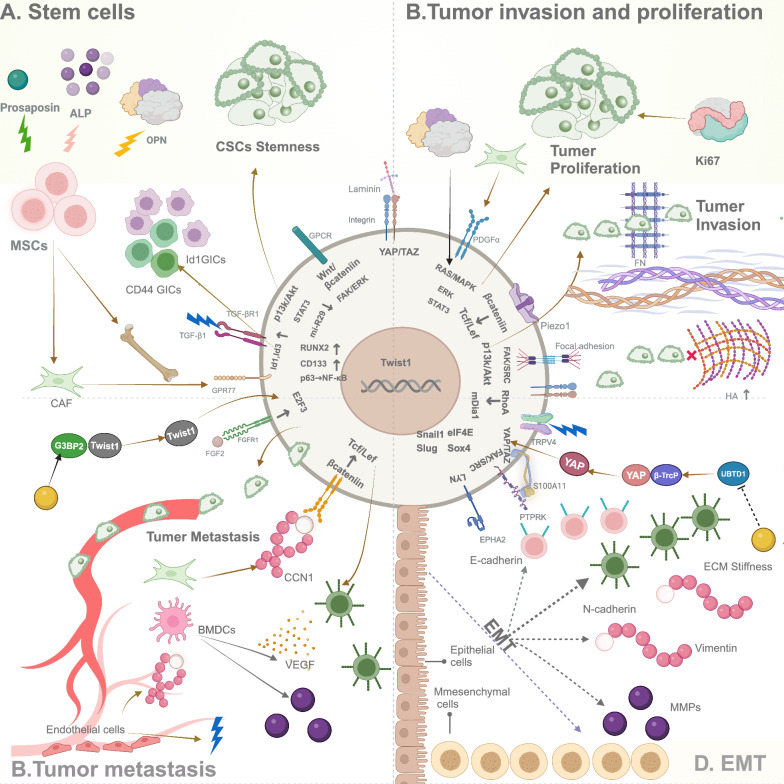
Regulation of ECM stiffness on CSCs, MSC, invasion, proliferation, metastasis, and EMT. **a**. ECM stiffness not only affects the stemness of CSCs but also upregulates the expression of Id1 and Id3 via the TGF-β pathway, enhancing the tumor-initiating capacity of GICs. Additionally, under high-stiffness conditions, MSCs can be induced to an osteogenic phenotype. **b**. ECM stiffness primarily promotes tumor invasion and proliferation by increasing and activating the FAK/SRC phosphorylation and PI3K signaling pathways. Besides ECM stiffness, the composition of ECM, such as collagen, HA, and FN, can also regulate the migration patterns of tumor cells. **c**. ECM stiffness promotes tumor metastasis by facilitating the nuclear localization of TWIST1 and the CD36-AKT-E2F3-FGF-2 pathway. Additionally, CAFs, ECs and BMDCs promote the colonization of cancer cells in distant organs by secreting growth factors, angiogenic factors, and MMPs. **d**. During EMT, the expression of N-cadherin, zinc finger transcription factor family members such as Snail, Slug, and Twist, and MMPs is upregulated, while the expression of E-cadherin is downregulated. ECM stiffness induces Snail expression through multiple signaling pathways, including S100A11 membrane translocation, eIF4E phosphorylation, and TGF-β1 autocrine signaling, thereby promoting EMT effects

Additionally, ECM stiffness also enhances cell proliferation efficiency by increasing the sensitivity of related protein expression [33]. For example, high stiffness ECM stimulates the proliferation of non-small cell lung cancer cells by enhancing the expression of Osteopontin (OPN) and thus upregulating the expression of integrin αVβ3 [33], which in turn activates FAK/AKT and ERK signaling pathways [34]. Notably, it has been shown that reducing ECM stiffness not only induces tumor cells to enter a resting state, but also reduces tumor cell spreading area, stress fibers, and focal adhesion, inhibits cell proliferation without inducing apoptosis, and exhibits stronger stem cell properties [29]. However, in some cases, lower stiffness can also promote the proliferation of cancer cells by inducing the expression of the proliferation-related protein Ki67 [32], which has important implications for tumor treatment strategies (Fig. 3b).

### Effect of ECM stiffness on tumors—invasion and migration

ECM stiffness is essential for the invasion and migration of tumor cells. Studies demonstrated that increased ECM stiffness promotes the invasive phenotype of tumor cells and accelerates tumor progression through a variety of signaling pathways, making them more aggressive. For example, the proliferation and invasiveness of the A549 lung adenocarcinoma cell phenotype were enhanced with increasing stiffness by evaluating the effect of ECM stiffness on cancer cell phenotype using an interpenetrating network (IPN) with different stiffness (30, 80, and 310 Pa). The results showed that in the lowest stiffness IPN, A549 cells formed organoid glands. However, in the highly rigid IPN, the cell clusters showed elongated shapes and the cells significantly invaded the matrix [35].

The response of tumor cells to ECM stiffness is regulated through multiple signaling pathways. Specifically, high matrix stiffness leads to increased activated 3D matrix adhesion formation and a consistently elevated outside-in/inside-out FAK-Rho signaling loop. This signaling network leads to overactivation of the Ras-MAPK pathway, which supports the growth and maintenance of an invasive phenotype of mammary epithelial cells in vitro and in vivo [36]. Alternatively, increased cross-linking of collagen leads to increased ECM stiffness, which contributes to the formation of focal adhesion and invasive bodies and enhances the invasiveness of tumor cells [37]. Mechanistically, LOX enhanced collagen cross-linking increases the stiffness of ECM, promotes the formation of focal adhesion, activates integrin β1, leads to increased phosphorylation of FAK/SRC and activation of PI3K signaling pathway, and ultimately promotes tumor cell invasion and tumor progression [38]. For example, breast cancer cells under high tension can enhance integrin-dependent mechanical signaling through the expression of FAK or p130cas, which are two integrin-adhesion focal proteins, and promote the progression of breast tumors [39].

Receptors from the G protein family, notably the small G proteins, play a vital role in the formation and characteristics of collagen fibers. G proteins enhance matrix stiffness by modifying the alignment of collagen. Increased matrix stiffness promotes the nuclear localization of the transcription factor Twist1 by decreasing the expression of its cytoplasmic binding partner, Ras-GTPase activating SH3 domain binding protein 2, thereby inducing invasion and metastasis of cancer [40]. Mechanistically, tumor cell migration is propelled by the high activity of Rho and Rac GTPases, and the specific migratory phenotype is determined by the activity of the dominant Rho family GTPases; The mesenchymal migration phenotype is primarily determined by Rac GTPase activity, while increased RhoA GTPase activity promotes ameboid migration. The oncogene Ras promotes the ameboid migratory phenotype by stimulating Rho activity, whereas the tumor suppressor p53 reduces tumor cell migration by decreasing RhoA activity [41].

In addition, TAGLN was identified as a potent mechanosensitive gene that mediates ovarian cancer cell migration and invasion in response to stiffness by regulating the RhoA/ROCK pathway in a feedback loop through binding to Src activation [42]. Overexpression of PIEZO1 promoted cell migration and invasion by enhancing the activity of integrin-FAK signaling pathway. In turn, the stiffer mechanical microenvironment enhances PIEZO1 expression, which promotes glioma invasiveness. Notably, localization of a subset of APC (adenomatous polyposis coli)-dependent RNAs is functionally critical for cell migration. Increased stiffness of the ECM promotes localization of APC-dependent Rnas. Thus, a detyrosinated microtubule network is formed that is essential for the localization of APC-dependent RNA (Fig. 3) [35].

The organization and composition of the ECM, not just density and stiffness, are critical for tumor cell migration and tumor progression. ECM components, like collagen, HA, and FN, not only affect the ECM stiffness but also directly control the migration pattern of tumor cells, including that increasing collagen matrix density increases ECM stiffness, thereby promoting an invasive phenotype. For example, noninvasive breast cancer cells grown in Matrigel, which contain nonfibrous collagen IV, acquire the capability for collective invasion when their culture substrate changes to fibrous collagen I, presumably because of fewer interactions with adhesion molecules that secure cells to collagen IV-rich basement membranes (BMs) [43].

In addition, CAFs are an important part of the TME, playing a role in enhancing tumor cell migration and invasion by adjusting the structure and physical characteristics of the ECM. Specifically, CAFs facilitate efficient and directed migration of cancer cells by generating FN-rich ECM with an anisotropic fibrous orientation. They further organize the FN matrix by augmenting nonmuscle myosin II and PDGFRα-mediated contractile and traction forces that are transmitted to FN through α5β1 integrin [44]. Additionally, in a mouse model of lung adenocarcinoma, collagen cross-linking mediated by PLOD2 expressed by CAFs was found to enhance tumor invasiveness.

Alternatively, alterations in the molecular weight of HA can notably influence cancer cell behavior, especially in reducing cell migration. Specifically, the elastic modulus, the size of pores in the collagen network, and the diameter of collagen fibers all increase with HA, but these pores are occupied with large HA molecules. Thus, high concentrations of HA reduced cell migration. Finally, in the BM, netrin-4 acts as a key regulator of BM stiffness. Netrin-4 softened the mechanical properties of native BM by opening the laminin node complex, reducing the potential of cancer cells to cross this barrier despite the formation of larger pores. Thus, the invasive activity of cancer cells was reduced, indicating that BM stiffness is a key factor affecting tumor metastasis. In summary, ECM stiffness drives tumor invasion and migration not only by directly altering the mechanical environment of tumor cells but also by modulating various matrix components (Fig. 3b) [38].

### Effect of ECM stiffness on tumors—metastasis

Cancer-related mortality is predominantly determined by metastasis rather than local tumor growth at the primary site. Contrasting with metastatic cancers, localized tumors can generally be cured through surgical intervention. Disseminated metastases often result in neurological dysfunction, respiratory failure, thrombosis, and other life-threatening complications. The process of metastasis necessitates cancer cells to surmount numerous biological hurdles, including escaping from the primary tumor, entering the circulatory system, colonizing distant organs, and proliferating. A multitude of factors contribute to the metastatic potential of cancer cells. Additionally, metastasis imposes distinct metabolic demands compared to those supporting cell proliferation, and inhibiting these metabolic pathways reduces metastatic dissemination [45].

ECM stiffness plays a pivotal role in cancer metastasis. Increased ECM stiffness, particularly in the liver, triggers the transformation of hepatic stellate cells into myofibroblasts, thereby enhancing their capacity to support tumor growth and metastasis [46]. Furthermore, heightened matrix stiffness stimulates the expression of LOXL2 in tumor cells via integrin β1/α5/JNK/c-JUN signaling pathways, promoting the production of FN and MMP9. These changes not only alter the physical properties of the TME but also facilitate the formation of pre-metastatic niches [27].

At the cellular level, ECM stiffness activates hepatic stellate cells through the CD36-Akt-E2F3 mechanical signaling pathway. The binding of E2F3 to the promoter of FGF2, confirmed by ChIP-qPCR, increases FGF2 expression, thereby promoting tumor proliferation and metastasis by activating FGFR1, PI3K/Akt, and MEK/ERK signaling pathways in HCC cells [47]. Moreover, the ECM protein CCN1/CYR61 is tightly regulated by mechanical strain in vascular ECs. Strain-induced CCN1 upregulates N-cadherin levels on the surface of ECs by promoting the nuclear translocation and signaling of β-catenin, thereby enhancing the crosstalk between cancer cells and ECs—a critical step in cancer metastasis [48]. Meanwhile, high matrix stiffness enhances the nuclear localization of TWIST1 by freeing it from its cytoplasmic binding partner G3BP2 [48], and induces EMT to promote tumor invasion and metastasis [49].

Furthermore, the activity of enzymes such as LOX not only enhances the cross-linking of collagen fibers but also promotes the colonization and growth of tumor cells in distant organs by activating related signaling pathways. For example, in breast cancer, collagen cross-linking at the primary site not only leads to cancer tissue sclerosis but also promotes adhesion plaque formation and PI3K/Akt signaling. ECM stiffness also recruits bone marrow-derived cells (BMDCs), which secrete growth factors, angiogenic factors, and MMPs to promote the colonization of cancer cells in distant organs [50].

In addition, other components and cell types in the TME, such as fibroblasts, ECs, and macrophages, also regulate the metastatic process of tumors by secreting various factors and participating in signaling pathways. For example, the expression of FN in Lewis Lung Carcinoma cells at the metastatic site promotes the aggregation of BMDC and the degradation of BM. The expression of periostin in fibroblasts is stimulated by tumor cells, which support the maintenance of cancer stem cells (CSCs). ECs in new blood vessels secrete periostin and TGF-β, which promote angiogenesis and metastasis. In addition, versican promotes metastatic growth by activating macrophages and establishing a proinflammatory microenvironment (Fig. 3c) [51].

### Effect of ECM stiffness on tumors—EMT

One of the most fundamental biological processes in tumor metastasis is the EMT process [52]. EMT is a two-way procedure whereby the epithelium is deprived of its properties and turns into mesenchymal cells, with changes in the expression of cell adhesion molecules and cytoskeleton. As a result, cells are endowed with enhanced mobility and invasion capabilities, which enable them to switch between epithelial and mesenchymal states in a highly dynamic and plastic fashion. The opposite process, termed mesenchymal-epithelial transition (MET), is commonly seen in developmental stages such as the development of the heart, renal morphogenesis, somatization, and cancer [53].

In biological systems, EMT plays a key role in many processes, including the development of embryos, inflammatory responses, fibrotic changes, tissue repair, and the progression of malignancies. EMT is characterized by a spectrum of changes, including the loss of apical polarity, enhanced anteroposterior polarity, reduced cell adhesion, and the adoption of mesenchymal traits. EMT is a continuous transformation in the EMT spectrum in which the cells lose their apical polarity, increase their anterior–posterior polarity, reduce cellular adhesion, change from epithelium to mesenchymal phenotype, and acquire mesenchymal characteristics. During EMT, the expression of mesenchymal factors such as N-cadherin, zinc-finger transcription factor families such as Snail, Slug, and Twist, and MMPs are up-regulated, and the expression of epithelial markers such as E-cadherin is decreased, which is associated with increased invasiveness and motility of tumor cells.

Increasing works have focused on the role of ECM stiffness in the EMT of cancer cells [54]. Researches indicate that higher matrix stiffness can induce EMT independently. The reason is that tumor tissues with higher stiffness not only highly express key molecules essential to EMT induced by stiffness [55] but also induce EMT by activating the FAK/Src signaling pathway and stimulating the expression of receptor tyrosine-protein phosphatase kappa [56]. Further studies revealed that Snail expression was involved in three signaling pathways involved in stiffening-mediated EMT effects, including the transfer of the S100A11 membrane and t. In addition, collagen itself also transmits signals to cells through receptors such as DDR1 and DDR2, and the activation of DDR2 stabilizes SNAIL1 and promotes the EMT process [48].

Meanwhile, high matrix stiffness results in TWIST1 nucleus localization and triggers EMT by weakening TWIST1 binding partner G3BP2 interaction [57] and promoting the activation of the EPHA2/LYN complex [58]. In other words, high ECM stiffness triggers the phosphorylation of the ligand-independent Ephrin receptor EPHA2, which in turn recruits and activates the LYN kinase. Once activated, LYN phosphorylates the EMT transcription factor TWIST1, releasing it from the cytosolic anchor protein G3BP2 and allowing it to move into the nucleus, thereby promoting EMT and invasion [59].

In addition to the above mechanisms, matrix stiffness also regulates PPIase non-mitotic alpha interaction 1 -dependent YAP activity through a non-Hippo mechanism and positively regulates the EMT process [60]. In addition, UBTD1 plays a role in the proteasome-dependent degradation of YAP by facilitating its interaction with the E3 ubiquitin ligase β-TrCP [61]. Matrix stiffness reduces the level of UBTD1 through CXCR4, thereby promoting the nuclear translocation of YAP.

TGF-β, the principal EMT inducer, stimulates the expression of SRY-Box transcription factor 4 (SOX4). This enables SOX4 to interact synergistically with Kruppel-like factor 5, driving the expression of transient receptor potential vanilloid 4 (TRPV4). TRPV4, as a mechanosensitive channel [62], is important for YAP/TAZ nuclear translocation and activation of AKT, which are key steps in EMT induced by matrix stiffness and TGFβ1. Inhibition of TRPV4 by genetic or pharmacological approaches blocks matrix stiffness and TGFβ1-induced EMT, Inhibition of TRPV4 through genetic or pharmacological means blocks matrix stiffness and TGFβ1-induced EMT, as evidenced by changes in cell morphology, adhesion, migration ability, and altered expression of EMT-related markers.

Interestingly, the high-stiffness microenvironment may also induce MET to help the mesh-like micrometastatic cells regain an epithelial phenotype and proliferate in the target organ. Studies have found that on a high hardness matrix (64 kPa) mimicking bone tissue, cells form multicellular clusters and relocate E-cadherin to the cell surface [63]. Similarly, additional studies have shown that softening of substrate material is able to promote certain features of EMT, including changes in the morphology of mesenchymal cells, increased expression of vimentin, and reduced expression of E-cadherin and β-catenin [31]. Taken together, these findings indicate that cells may respond differently to ECM stiffness in different cell states. For example, before metastasis, cells may acquire mesenchymal features through EMT, while in target organs, they regain epithelial features through MET. Therefore, it is important to investigate the regulatory mechanisms of ECM stiffness on EMT and MET for understanding cancer metastasis and therapy (Fig. 3d).

### Effect of ECM stiffness on tumors—angiogenesis

Angiogenesis refers to the process of establishing a new blood vessel network through the migration, proliferation, and tube formation of ECs. Under normal conditions, angiogenesis is tightly regulated, but in the TME, the formed tumor blood vessels are usually disorganized, leaky, and inefficient, leading to inefficient drug delivery and loss of therapeutic effect [64]. Matrix stiffness plays a role in angiogenesis, which regulates the formation and function of tumor blood vessels through a variety of mechanisms.

Increased ECM stiffness significantly alters the mechanosensing and signaling pathways of ECs, of which YAP/TAZ signaling pathway is a key regulatory mechanism. YAP/TAZ, as a key transcriptional coactivator in the Hippo signaling pathway, is activated upon increased ECM stiffness and moves into the nucleus to promote the transcription of genes related to angiogenesis [65]. In addition, ECM with increased stiffness provides a sturdier support for ECs, promoting their migration and proliferation, and further driving angiogenesis through activation of key signaling pathways such as PI3K/AKT, MAPK, and RhoA activity [52]. At the same time, with the change in stiffness, the internalization of VEGF by ECs increases, which directly affects the activation of AKT and ERK signaling pathways and accelerates the proliferation, migration, and survival of ECs [66]. Mechanistically, COL1-enhanced tissue stiffness increased Piezo1 expression through miR-625-5p. The expression of Piezo1 can stimulate the expression and secretion of VEGF, CXCL16, and IGF binding protein 2. In addition, Piezo1 activation due to matrix stiffness inhibits HIF-1 ubiquitination, thereby enhancing the expression of downstream pro-angiogenic factors and accelerating EC formation [67].

Interstitial myofibroblasts may express more proinflammatory cytokines and chemokines, such as IL-8, and TNF-α, in an environment of increased stiffness. These inflammatory factors can recruit immune cells, such as macrophages, neutrophils, and mast cells, which further secrete VEGF, thereby indirectly promoting angiogenesis. In addition, stromal myofibroblasts also secrete more MMPs, such as MMP-1, MMP-2, MMP-3, MMP-7, MMP-11, and MT-MMP1. MMPs can degrade the matrix components, promote EC migration, and provide space for creating a new vascular system [68]. In addition, increased ECM stiffness may be associated with specific gene mutations in interstitial myofibroblasts, such as mutations in TP53 and PTEN, which lead to excessive deposition of ECM components and increased stiffness, further exacerbating angiogenesis [68].

ECM components such as collagen, TNC, FN, and HA significantly affect the behavior of ECs. Specifically, ECM stiffness is increased through type I collagen, forming a physical barrier limiting EC migration and invasion. TNC induces angiogenesis and vascular permeability by down-regulating DKK1 and increasing Wnt signaling [110]. In addition, focal adhesion composition and conjugation to Fn are also altered by stiffness, ECM stiffness enhances the Arg-Gly-Asp (RGD) binding site on Fn, and synergism site, which may result in an increase in αvβ3 integrin binding and decreased α5β1 engagement. Thus, angiogenesis is promoted [66].

TME with low-molecular-weight (LMW) HA is related to the promotion of angiogenesis by expressing pro-angiogenic factors such as VEGF-A, VEGF receptor 2 (VEGFR-2), and HA binding protein-1. For example, The conjunction of doxorubicin and LMW HA enhanced EC migration and the development of structures resembling blood vessels [64]. HA has the capacity to modulate the binding interactions of surface polyanions on membranes, as in breast cancer cells, the addition of LMW HA leads to the release of fibroblast growth factor 2 (FGF-2) from heparan sulfate proteoglycans, which in turn increases ECs migration and angiogenesis, independent of VEGF. In addition, LMW HA stimulates the expression of MMPs, thereby favoring tumor cell invasion and angiogenesis [69].

**Fig. 4:**
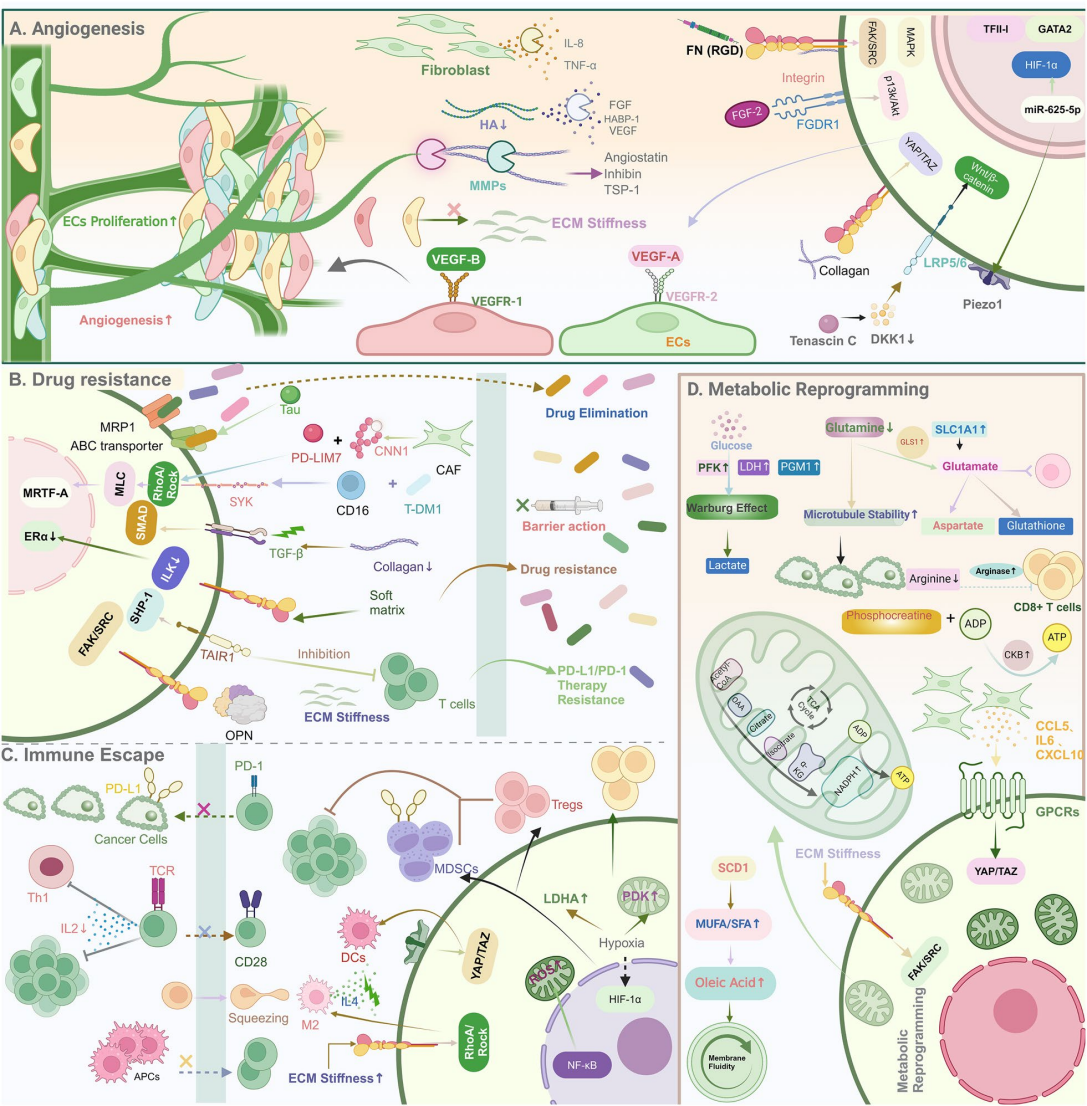
Regulation of ECM stiffness on Angiogenesis, Drug Resistance, Immune Escape and Metabolic Reprogramming. **a**. ECM stiffness supports the growth and survival of ECs mainly by enhancing the expression of VEGF and its receptors; On the contrary, ECM stiffness can also act as a barrier to ECs migration, and ECM protein fragmentation can also act as an anti-angiogenic agent. **b**. Matrix stiffness actively removes chemotherapeutic drugs from cancer cells by enhancing the functional activity of MRP1, ABC transporters on the cell membrane, and also acts as a barrier to block drug delivery **c**. EC stiffness not only acts as a physical barrier to block the infiltration of immune cells into the TME, but also limits the contact of anti-apcs with T cells and regulates the polarization of macrophages. **d**. Matrix stiffness regulates glycolysis, amino acid metabolism, and lipid metabolism in tumors through multiple mechanisms

Overall, tumors stimulate the formation of a new vascular system for supplying oxygen and nutrients needed for growth and survival. Angiogenesis, driven by factors like VEGF and FGF, comprises the migration and proliferation of ECs within nutrient-deficient tissues, especially in regions adjacent to tumors, followed by their assembly into functional blood vessels. The ECM serves as a storehouse of proangiogenic and antiangiogenic factors, provides access for ECs migration, and promotes the growth and survival of newly recruited ECs. Tumor-associated ECM with enhanced stiffness promotes angiogenesis through enhancing ECs migration and activation of GATA2 and TFII-I transcriptional pathways, enhancing VEGFR-2 receptor expression and supporting ECs growth and survival. However, high ECM stiffness may also negatively impact the integrity of blood vessels, trigger MMP activity, and inhibit angiogenesis by releasing anti-angiogenic factors [41], specifically, ECM protein cleavage-produced fragments including endostatin, oncostatin, angiostatin, inhibin, and Thrombospondin-1 have anti-angiogenic functions. These fragments are derived from type IV and XVIII collagen and block neovascularization by binding to cellular receptors such as integrins and EGFR to inhibit EC proliferation, migration, and lumen formation. In particular, inhibins inhibit angiogenesis by binding to α1β1 integrins and antagonizing MAPK signaling (Fig. 4a) [51].

### Effect of ECM stiffness on tumors—drug resistance

Increased matrix stiffness significantly affects drug delivery and cellular resistance through multiple signaling pathways. For example, enhanced matrix stiffness enhances chemoresistance of tumor cells through the Src and ERK/MAPK pathways or FAK/Src/β-catenin signaling axis. Enhanced matrix stiffness enhances EMT markers in pancreas and breast carcinoma, and facilitates YAP and TAZ nucleus localization, resulting in resistance to chemotherapy agents such as paclitaxel (PTX) and doxorubicin [70]. Meanwhile, studies have shown that high matrix stiffness markedly impairs metformin’s ability to inhibit liver cancer invasion and metastasis. Mechanically, mechanical stiffness signaling activates PTEN/PI3K/Akt/MMPs and inactivates metformin, which is jointly involved in metformin resistance induced by matrix stiffness [71]. In BRAF-mutant melanoma cells, ECM stiffness activates YAP/TAZ, thereby significantly reducing the responsiveness of the cells to specific drugs such as vemurafenib and lapatinib [5]. In addition, Trastuzumab interacts with CD16, which results in increased contractility of cells by activating the SYK-VAV2-RhoA-ROCK-MLC2-MRTF-A pathway and increasing matrix production, thereby reducing drug delivery [70].

In addition, matrix stiffness can actively expel chemotherapy drugs from cancer cells by enhancing the functional activity of multidrug resistance protein 1 (MRP1) on the cell membrane, thus making cancer cells resistant to chemotherapy [72]. Meanwhile, ECM stiffness significantly affects the activity of ABC, which determines the uptake of intracellular drugs. In pancreatic, HCC, and breast cancer cells, matrix stiffness leads to resistance to 5-Fu, cisplatin, PTX, doxorubicin, and mitoxantrone by upregulating ABC drug transporter gene expression. Similarly, Jurkat and HSB2 leukemia cells, which are grown on the collagen matrix, become doxorubicin resistant by activating integrin/Erk signaling and up-regulating the ABCC1 transporter. Some studies have suggested that soft substrates may lead to a reduction in ABC transporter gene expression and activity, which reduces drug resistance, leading to enhanced drug clearance and improved treatment outcomes [70].

Collagen is an important part of the ECM, and increased collagen content leads to drug resistance in cancer therapy through various signaling pathways. For example, in esophageal squamous cell carcinoma, increased collagen content leads to chemotherapy resistance through the MAPK and PI3K/AKT pathways. However, the accumulation of collagen and HA in pancreatic cancer enhanced the resistance to doxorubicin. Cross-linking of collagen at the transfer site increases tissue stiffness and promotes treatment resistance. In addition, different types of collagen exhibit different resistance mechanisms, such as COLI through activation of β1 integrin and multiple signaling pathways, and COLI and ColVI-induced tau upregulation leading to resistance to multiple drugs [40]. In lung tumors, increased collagen induces T-cell exhaustion through the activation of SHP-1 signaling pathway by the LAIR1 receptor, leading to resistance to anti-PD-1/PD-L1 immunotherapy [73]. Furthermore, in pancreatic ductal adenocarcinoma, collagen IV depletion leads to resistance to VEGF therapy through TGF-β signaling [40].

In addition to collagen, CAFs contribute to gastric cancer resistance to 5-Fluorouracil (5-Fu) by secreting myosin light chain 1 (CNN1). Mechanically, CNN1 interacts with PDLIM7 to prevent its degradation through NEDD4-1, which activates the ROCK1/MLC pathway, leading to increased matrix stiffness and enhancing 5-Fu resistance in gastric cancer cells via YAP activation [74]. In addition, specific proteins such as OPN play a key role in EGFR-TKI resistance by activating the integrin αVβ3/FAK pathway in non-small cell lung cancer [75]. Inhibiting FAK signaling may increase the susceptibility of PC9 gene resistance to EGFR-TKI in vitro and in vivo experiments [34].

Although in general, increased matrix stiffness is correlated with drug resistance, in certain cases, breast cancer cells in a soft microenvironment (resembling metastatic sites like bone marrow, brain, and lung) exhibit heightened tamoxifen resistance. This phenomenon is attributed to the soft matrix, which triggers autophagy through integrin-linked kinase (ILK) and decreases estrogen receptor-α (ERα) expression. Pharmacological inhibition or gene down-regulation of autophagy can be a strategy for overcoming drug resistance in breast cancer (Fig. 4b) [76].

### Effect of ECM stiffness on tumors—immune escape

The immune system serves as a critical regulator of tumor biology, supporting or inhibiting tumor progression, growth, and invasion, and delivering drug therapeutic effects. On the tumor side, they have developed mechanisms to evade the action of the immune system, ranging from direct secretion of biochemical signals to indirect responses, in which the cellular participants of the TME cooperate to mechanically regulate the ECM to make it unsuitable for immune cells [77]. ECM stiffness is a critical factor in tumor biology, offering structural support to tumor cells and simultaneously impacting tumor progression and therapy outcomes through the regulation of immune cell migration, localization, and functionality [78].

ECM stiffness and composition act as a physical barrier [78] to hinder the infiltration of immune cells into the TME through a variety of physical and chemical mechanisms, and fine-regulate the migration and localization of immune cells. For example, the ability of T cells to mount an antitumor response depends on the balance of a network of reticular fibers that promote migration against regions of unfavorable migration composed of dense and ordered ECM structures [15]. In general, T cells prefer to pass through thin fibrous regions rich in collagen and FN and avoid the denser matrix formed by Lys oxidase cross-linking. Inhibition of LOX can weaken the fibrillar collagen network and improve the pathway of T cells into the tumor [79]. Moreover, in immune checkpoint therapy, the dense ECM acts as a physical obstacle that limits T-cell infiltration and reduces PD-1/PD-L1 interactions [80], thereby limiting the cytotoxicity of cancer cells. When T cells traverse through high-density collagen fibers leads to nuclear damage, reduced motility, and ultimately cell death.

Increased matrix stiffness enhances PD-L1 expression in cancer cells [81], which was associated with nuclear translocation of YAP/TAZ. Thus, when ECM remodeling and stiffness are targeted using LOX, T-cell migration is enhanced, and the effectiveness of anti-PD-1 blockade is improved [82]. In line with this, the mechanical therapy tranilast, combined with immune checkpoint inhibition, helps restore TME abnormalities, in combination with inhibition of the immune checkpoint, increased T-cell infiltration in the TME and immune memory in breast cancer mice resistant to immunotherapy [83]. In addition, the tight structure of the ECM may limit the access of antigen presenting cells (APCs) to T cells and reduce the opportunity for antigen presentation.

ECM stiffness also changes intracellular signal transduction pathways through mechanical stress, especially the integration of costimulatory signals involving TCR and CD28, resulting in the inability of T cells to produce IL-2 effectively, thereby weakening the CD3—and CD28-mediated activation and proliferation of T cells and affecting their differentiation into Th1 cells [41]. In other words, within a certain range of stiffness, T cell proliferation and IL-2 secretion will increase first, reach a peak, and then decrease as the stiffness continues to increase [84].

ECM can also bind to LAIR receptors through type I collagen. It directly inhibits T cell proliferation and activation from the naive state [33]. Meanwhile, ECM stiffness can regulate integrin-mediated cell binding and biochemical signaling through mechanical tensile properties. For example, in the ECM with low stiffness, dendritic cells can migrate in an amoeboid fashion on a scaffold of type I collagen fibers, independent of the integrins β1, β7, β2, and αv. Other immune cells, such as CD4 and CD8 T-cell blasts and monocyte lines, can also undergo protease and integrin β1 independent migration through type 1 collagen gels [80].

ECM stiffness not only acts as a physical barrier to hinder the infiltration of immune cells into the TME but also promotes the survival and proliferation of tumor cells by activating specific signaling pathways [85]. For example, matrix stiffness can not only regulate macrophage polarization by regulating YAP expression and nuclear localization [86] but also regulate mitochondrial ROS production through the Rho pathway, which in turn triggers the NF-κB pathway to regulate macrophage polarization. For example, macrophages cultured in a softer substrate, when stimulated with lipopolysaccharide/ATP, showed higher ROS production, higher CD86 expression, NO release, and round cell morphology with faster migration rate, and secretion of more pro-inflammatory factors (e.g., IL-1β, TNF-α). It promotes the polarization of classically activated M1 macrophages, thereby enhancing the pro-inflammatory response [87]. On the contrary, at moderate matrix stiffness, macrophages express more CD206, produce less ROS, secrete more anti-inflammatory factors, and promote the polarization of alternative activated M2 macrophages, thereby inhibiting the inflammatory response [88].

ECM stiffness also regulates the function, maturation, metabolism, and phenotype of dendritic cells (DCs) through a variety of mechanisms. DCs cultured at physiological stiffness showed lower proliferation, activation, and cytokine production, whereas DCs cultured at higher stiffness showed enhanced activation and metabolic flux. Mechanistic studies confirmed that ECM stiffness enhances the activity of DCs by activating TAZ and calcium channels (such as PIEZO1) in the Hippo signaling pathway, thereby triggering an adaptive immune response [89].

High ECM stiffness acts as a physical barrier, hindering angiogenesis and oxygen diffusion, which consequently results in localized hypoxia. Furthermore, high ECM stiffness promotes glycolysis (Warburg effect), thereby stimulating lactate overproduction and exacerbates acidosis and hypoxia microenvironment by activating the integrin-FAK-YAP pathway, while higher ECM stiffness can regulate the metabolic function of T cells through hypoxia [84]. Under hypoxia, the transcription factor HIF dissociates from its negative regulator, von Hippel-Lindau, and thus upregulates its target genes to stabilize Hif-1α and increase the expression of pyruvate dehydrogenase kinase and lactate dehydrogenase A, thereby reducing oxidative phosphorylation. The anti-tumor activity of CD8 + T cells was enhanced [78]. In addition, hypoxia upregulates PD-L1 on myeloid-derived suppressor cells (MDSCs) via HIF-1a, leading to T cell exhaustion and possibly promoting the generation of regulatory T cells, thereby exerting immunosuppression (Fig. 4c).

### Effect of ECM stiffness on stemness

Cancer is a highly heterogeneous disease containing multiple phenotypically and functionally distinct cell subpopulations. The properties of CSCs are characterized by features resembling those of normal stem cells, which are crucial in the progression of tumors. A minority of cancer cells have stem-like characteristics, enabling them to self-renew and initiate tumor formation. These cells are regarded as major factors driving tumor progression, drug resistance, disease recurrence, and metastasis [90].

ECM stiffness is crucial to CSCs’ biology function regulation, which transmits signals from the microenvironment through mechanical stiffness. In general, higher matrix stiffness can promote the expression of CSC-like phenotype and reduce apoptosis induced by chemotherapy drugs (e.g., sorafenib) [32]. Mechanically, high stiffness matrix enhances the expression of CSC stemness markers including CD133, ALDH1, and Lgr5 by activating YAP/TAZ, FAK/ERK, Wnt/β-catenin, and CXCR4 pathways. In addition, stiffness can also sense receptors to activate Ras, Rac, MAPK, and PI3K signaling pathways, increasing cell proliferation and stem cell characteristics [91].

Matrix stiffness can also influence MSCs’ differentiation orientation. Under high stiffness conditions, MSCs can be induced to an osteogenic phenotype and produce significantly high levels of RUNX2, alkaline phosphatase (ALP), and OPN, and the cell morphology is polygonal and spreads over a larger area [92], while the lower stiffness matrix promotes adipocyte differentiation [93]. For example, on the rigid ECM, MSC has more long-distance chromatin interactions but fewer A-domains, which are normally associated with active chromatin. The transition from compartment B on the soft ECM to compartment A on the rigid ECM is associated with genes that code for proteins responsible for cytoskeletal organization. At the level of Topologically Associated Domains (TAD), the TAD on rigid ECM tended to be more merged than those on soft ECM, and these merged TAD contained many up-regulated genes encoding osteogenic proteins, such as SP1, ETS1, and DCHS1. The up-regulation of these genes has been confirmed through quantitative real-time polymerase chain reaction, and was consistent with the increase in ALP staining, indicating the differentiation of the cells towards osteogenesis [15]. In addition, the increase of ECM stiffness in the TME can also induce MSC to differentiate into CAF and secrete the signaling protein prosaposin to realize the transduction of mechanical signals in MSCs, thereby triggering the proliferation and survival ability of cancer cells [94].

While high ECM stiffness promotes stem cell properties and increases cell proliferation in most cases [95], soft environments induce cell quiescence and enhance the expression of stem cell markers in HCC, including CD44, CD133, c-kit, CXCR4, OCT4, and NANOG [29]. For example, HCC cells in a soft matrix showed small and round shapes and stemness properties and increased expression levels of liver CSC surface markers, as well as the increased number of side population cells. The soft matrix induces early G1 phase arrest of the cell cycle and enhances the ability of spheroid formation [33]. Meanwhile, with the decrease of matrix stiffness, miR-29 significantly down-regulated and activated PI3K/Akt and Stat3 signaling pathways, and the expression of stemness-related transcription factors and sphere formation ability of osteosarcoma cells were enhanced, while the expansion area, proliferation, and migration were inhibited [96]. Mechanistically, HCC is often accompanied by a cirrhotic background, fibrosis and soft nodules coexist, and soft regions may be enriched for CSC niche [97]. As for signaling pathways, YAP/TAZ downregulates miR-29 in the soft ECM through non-classical pathways (e.g., miR-29/PI3K/Akt), disinhibits PI3K/Akt pathway, activates downstream STAT3, and drives the expression of stem-like genes in HCC, which is different from the stiff-YAP positive feedback in most cancers [95]. In addition, soft matrix reduced cytoskeletal tension and inhibited Hippo kinase (LATS1/2), resulting in reduced YAP/TAZ nuclear translocation [29], but maintained stemness through compensatory β-catenin activation via PI3K/Akt pathway [29].

In different types of cancer, ECM components such as collagen type I, laminin, and HA not only affect the self-renewal and proliferation of CSC but also enhance its resistance to chemotherapy drugs and metastatic ability [70]. CSC enrichment and maintenance were promoted through different signaling pathways, such as Hippo, FAK, Akt/mTOR/YAP, and Twist-TGFβ-snail. These ECM components, such as laminin, form a stem cell matrix by interacting with TAZ, a transcriptional regulator of the integrin and Hippo pathway, as well as Wnt and Notch signaling pathways, and promote CSC’s stemness through a feedback mechanism. In addition, laminin induces the differentiation of stem cells. For example, specific laminin isoforms such as laminin-111 play a key role in promoting stem cell differentiation and exit from pluripotency, as demonstrated by in vivo and in vitro experiments [98]. Secondly, CD10 + GPR77 + CAFs maintain phosphorylation and acetylation of p65 through complement signal mediated by GPR77 (C5a receptor), and then continuously activate NF-κB, maintain the survival site of CSCs, and promote cancer formation and chemoresistance [99]. Other ECM molecules, such as chemokines, cytokines and adhesion molecules, also play important roles in regulating CSC niche homeostasis. Finally, TGFβ is also essential for inducing CSC phenotype [70], and by inhibiting the TGF-β pathway and down-regulating Id1 and Id3 expression, CD44high and Id1high GIC groups are reduced, thereby reducing the tumorigenic ability of GICs (Fig. 3a) [100].

### Effect of ECM stiffness on tumors—metabolic reprogramming

Metabolic reprogramming, a hallmark of malignancy, was first identified a century ago. Metabolic reprogramming in cancer cells not only supplies energy but also produces critical metabolites essential for biosynthesis, sustained proliferation, and tumor development. Important metabolic pathways, such as aerobic glycolysis, glutaminolysis, macromolecular synthesis, and redox homeostasis, are crucial for cancer cell progression [7]. In certain scenarios, altered metabolic activity resulting from reprogramming can be utilized for diagnosing, monitoring, and treating cancer [101].

ECM stiffness can regulate glycolysis, amino acid metabolism, and lipid metabolism by various mechanisms. Firstly, ECM stiffness leads to higher expression of critical glycolytic enzymes such as phosphofructokinase (PFK) and lactate dehydrogenase (LDH) by activating the YAP/TAZ signaling pathway [102]. In addition, CAFs regulate the metabolic reprogramming of tumor cells by secreting cytokines such as CCL5, IL6, and CXCL10 in the rigid ECM environment. These cytokines promote glycolysis by increasing the expression of PFK and LDH. It increases glycogen mobilization through phosphorylation of Phosphoglucomutase 1 (PGM1). Moreover, by activating the TCA cycle, increasing NADPH synthesis and oxidative phosphorylation, it regulates the metabolism of cancer cells and enhances tumor cell proliferation in vivo [45]. Second, ECM stiffness promotes the expression of glutamate transporters (SLC1A1 and SLC1A3) and Glutaminase 1, resulting in elevated glutamate uptake and production. Glutamate is used to produce glutathione and aspartate, which promotes REDOX balance, the synthesis of nucleotide, and eventually ECM remodeling.

Glutamine depletion caused by ECM stiffness increases microtubule stability and enhances cancer cell proliferation and invasive migration. In macrophages, increased ECM stiffness results in reprogramming of arginine/proline metabolism and secretion of arginase, which impairs the antitumor activity of CD8^+^ T cells [103]. In addition, a rigid environment promotes creatine phosphorylation through YAP-dependent increased cytoplasmic creatine kinase type B (CKB) expression. Finally, ECM stiffness promotes cancer invasion and metastasis by reprogramming lipid metabolism in tumor cells and improving cell membrane mobility. Mechanistically, as the stiffness of the ECM increases, mechanical signals down-regulate ubiquitin–proteasome degradation of stearoyl-CoA desaturase 1 (SCD1) by activating integrin β1/FAK signaling, leading to increased SCD1. Increased SCD1 acts as a mediator of fatty acid desaturation, increases monounsaturated fatty acid (MUFA)/saturated fatty acid (SFA) ratio, and especially increases oleic acid production, which improves cell membrane fluidity and ECM stiffness-mediated cell invasion (Fig. 4d) [102].

### Effect of ECM stiffness on tumors—genomic stability

Genomic stability refers to the ability of cells to maintain the integrity and accuracy of genetic information during DNA replication, damage repair, and cell division. The key mechanisms include DNA damage repair, replication fidelity, cell cycle checkpoint regulation, telomere maintenance, and epigenetic regulation (chromatin structure stabilization). Abnormal genomic stability can lead to the accumulation of mutations and chromosomal aberrations, which are closely related to cancer, aging and genetic diseases [104]. Microfluidic technology was used to simulate the interstitial pores through which cancer cells pass during the invasion, and it was found that when tissue stiffness increased, cells experienced a temporary rupture of the cell nuclear membrane, accompanied by DNA damage [105]. Mechanistically, the pore size of the matrix is decreased by overdepositing of matrix proteins like collagen. When cancer cells invade, they must be squeezed through narrower pores and undergo more physical damage [97]. This extrusion motion would allow some mobile proteins to be separated from DNA205 [106]. For example, DNA repair proteins like BRCA1, thereby enhancing the potential for genome integrity [8]. This finding suggests that increased tissue stiffness may trigger a mechanical stress response in the nucleus during cell invasion and may have an impact on the genetic stability of the cell. Furthermore, matrix stiffness promotes cell mitosis, during which spontaneous mutations are accumulation alongside rapid DNA replication. Furthermore, matrix stiffness increases the probability of nuclear envelope breakdown [107], which allows nuclear contents such as nucleic acids and nucleases to leak into the cytoplasm, eventually causing DNA damage and genome integrity.

ECM stiffness regulates the efficiency of DNA double-strand break (DSB) repair and cellular sensitivity to genotoxic agents through the MAP4K4/6/7 kinase-dependent phosphorylation of ubiquitin. DSBs constitute highly deleterious lesions that underlie genetic instability, affecting the efficacy of radiotherapy and many cancer chemotherapy drugs. Lower ECM stiffness reduces DSB repair efficiency, making cells more sensitive to genotoxic drugs. Mechanistically, low ECM stiffness activates MAP4K4/6/7 kinase, leading to the phosphorylation of ubiquitin.

## Matrix components as therapeutic targets

**Fig. 5:**
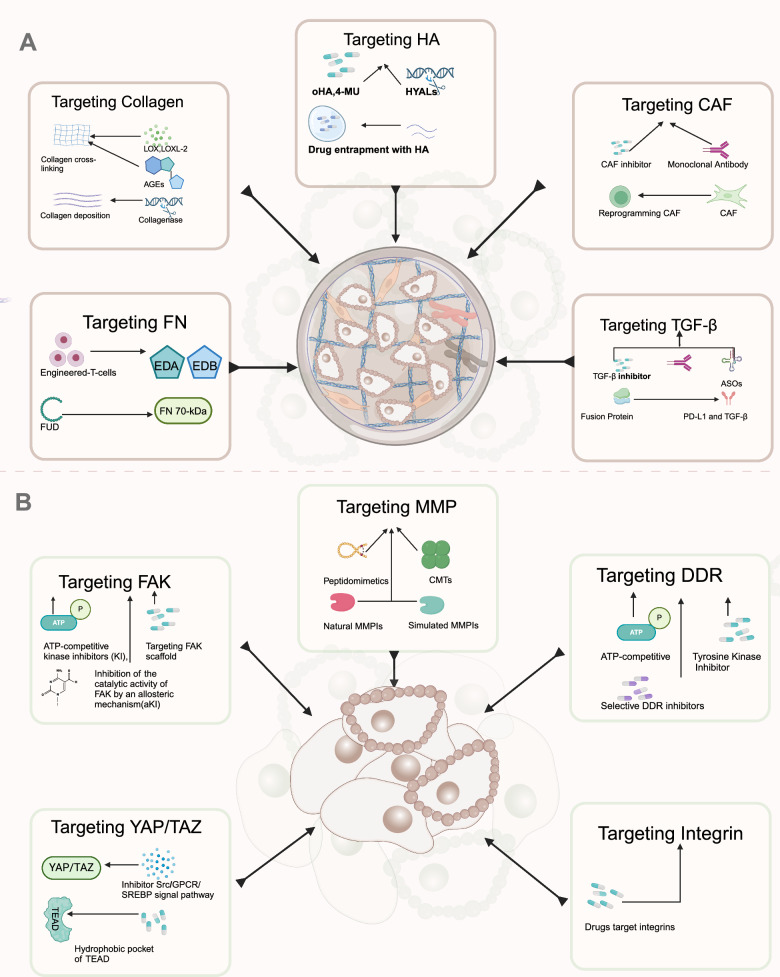
The diagram of ECM-targeting treatment. **a**. Targeting ECM components to reduce matrix stiffness, thereby improving drug delivery and enhancing the infiltration of immune cells within the tumor. **b**. Blocking downstream receptors associated with ECM stiffness to treat tumors

Targeting ECM stiffness may be a promising approach for cancer treatment and drug resistance. In addition to promoting tumor formation, ECM stiffness prevents therapeutic agents from penetrating the tumor interior and limits immune cell diffusion in the tumor [108]. At the same time, the elevated tumor interstitial fluid pressure within the tumor decreases to baseline levels at the periphery, resulting in an outward flow of fluid from inside the tumor to surrounding tissue, which washes the drug away from the tumor. In solid tumors, ECM stiffness depends mainly on the component of ECM and the structure of the tissue, while at the tissue level, ECM stiffness is mainly determined by compression and abnormality of tumor vasculature and accumulation of matrix composition [70]. Thus, tumor stiffness and mechanical forces can be reduced by targeting ECM components, hence facilitating tumor perfusion and drug delivery. The mechanisms of this therapy and the progress in clinical studies will be discussed in the following sections (Fig. 5a).

### Therapies targeting collagen

Collagen occupies a central position in the construction of ECM and provides a “channel” for cancer cell migration and invasion in some particular collagen tissues. In order to solve the issue of overaccumulation of collagen in TME sclerosis, a number of treatments have been proposed, which are primarily concerned with the synthesis, degradation, and cross-linking of collagen.

TGF-β has a significant effect on collagen synthesis, and TGF-β is a potential target for inhibition of collagen production. Ajulemic acid, a synthetic cannabinoid derivative, has demonstrated efficacy in inhibiting TGF-β production and exerting anti-inflammatory effects. Its mechanism of action is to activate cannabinoid receptor 2, leading to the production of prostaglandins, which reduce inflammatory cytokines and fibrosis [109]. Meanwhile, other drugs such as Halofuginone and fresolimumab have been demonstrated to decrease the production of collagen through inhibition of TGF-β signaling. These drugs are currently undergoing evaluation in several clinical trials (NCT01401062 and NCT02581787) for cancer treatment [8].

Nonetheless, therapeutic targets involving TGF-β ought to be treated with caution because of potential long-term side effects in the context of long-term use [110]. Apart from TGF-β, a number of experimental trials have demonstrated the efficacy of angiotensin-II receptor agonists, such as losartan, which are commonly used to treat hypertension, and have been shown to be effective in the treatment of hypertension. However, they also inhibit TGF-β signaling [21], CCN2 and ET-1 expression, and effectively inhibit type I collagen synthesis and deposition (NCT01821729 and NCT04106856) [91], while improving the penetration and therapeutic efficacy of intratumoral and intravenous delivery nanoparticles (Doxil and HSV) [102].

In addition to losartan, collagenase, as an enzyme capable of degrading collagen, has shown significant effects in reducing matrix stiffness and promoting effective drug delivery to solid tumors [8]. In a mouse model, treatment with liposome-encapsulated collagenase was even achieved, with an 87% reduction of malignant tumors. However, this treatment should be considered with caution and is best applied in early-detected cancer cases with significant matrix stiffness and no signs of invasion or metastasis [91].

In addition to the excessive accumulation of collagen leading to fibrosis, another significant alteration is the increased cross-linking, which affects the stability of the collagen network and leads to irreversible fibrosis [111]. The synthesis of collagen cross-linking is caused by the conversion of certain Lys and Hyl residues to their corresponding aldehydes, a reaction catalyzed by LOX and LOXL proteins. Inhibition of LOX and LOXL2 has been suggested as a promising therapy [26].

BAPN is a natural compound first discovered to inhibit LOX [112]. In recent years, researchers have developed a series of new synthetic compounds that can act as selective or dual inhibitors of LOX/LOXL2. PXS-S1A is a first-generation LOX inhibitor with comparable activity and selectivity to BAPN [105]. In addition, PAT-1251 is the first irreversible LOXL2 inhibitor small molecule developed by PharmAkea (San Diego, CA, USA), a compound that is a potent and highly selective oral LOXL2 inhibitor based on benzylamine and the 2-substituted pyridin-4-ylmethylamine, Although PAT-1251 was found to be well tolerated and successfully passed Phase I clinical trials (NCT02852551), no Phase II clinical trials of PAT-1251 have been completed to date [113]. In vivo preclinical studies of the aminoethiopene-based LOX inhibitor CCT365623 demonstrated that inhibition can delay the progression of tumors and decrease pulmonary metastasis in a mouse breast cancer model [114].

In addition to small-molecule inhibitors, another LOXL2 inhibitor is the patented de primary amino-containing diazocynonane compound, which was tested in a transgenic mouse model of breast cancer and shown to availably reduce lung metastasis formation. Another LOXL inhibitor a transgenic mouse model of breast cancer has shown an anti-LOXL2 monoclonal mouse antibody, AB0023, which has shown efficacy in lowering intratumoral collagen-like density in a rat pancreas carcinoma model, Subsequently, a humanistic form of this antibody, simtuzumab, has been developed to inhibit allosteric activity by binding to the fourth SRCR domain of LOXL2, a non-competitive extracellular LOXL2 inhibitor. This antibody has shown improved mouse survival in various preclinical models of fibrosis and cancer. However, in clinical trials, the combination of simtuzumab and gemcitabine did not effectively enhance the treatment outcome of patients with pancreatic cancer [100].

Additional anti-LOXL2 antibodies, such as GS341, have been developed in mice, leading to decreased cross-linking of collagen in hepatic fibrosis [114]. However human versions of the antibodies have not been developed, so it remains unclear whether they will be able to achieve similar improvements in human survival as observed in animal models. Other studies have found significant effects with the potent blocking of TGF-β1 response, Snail1 expression, and collagen deposition by trihydroxyphenol compounds in models of pulmonary fibrosis and collagen-dependent lung cancer metastasis. Its mechanism of action depends on the presence of active LOXL2, which irreversibly inhibits LOXL2 by triggering the autoxidation of LOXL2/3 specific Lys, and converts to a new metabolite that directly inhibits TβRI kinase. This double inhibition is effective in preventing pathologic collagen-related accumulation but does not have the toxic effect of global inhibitors [115].

Another oral copper chelator that interferes with collagen cross-link formation is D-penicillamine. It has been shown that D-penicillamine is able to complexate Lys-derived aldehydes, making them unable to form cross-links [105]. D-penicillamine has been used to treat diseases with collagen accumulation, such as liver fibrosis and progressive systemic sclerosis [116]. Furthermore, in order not to cause secondary conformational changes in the protein, Mohankumar et al. developed ‘M’peptides designed to bind to the Cu-binding region of LOX through competitive inhibition. The peptides decreased the extracellular LOX activity in the human umbilical vein endothelial cells conditioned culture, but no in vitro or in vivo tumor trials were conducted. Furthermore, it has been demonstrated that inhibition of the usage of copper transport agents. For example, antioxidant 1 copper chaperone and superoxide dismutase copper chaperone can decrease the LOX activity outside the cell.

In addition to LOX-catalyzed generation, collagen cross-linking can also occur through non-enzymatic pathways, such as the formation and accumulation of AGEs [117], which are formed by the reaction between the reducing carbonyl groups of sugars and the free ε-amino groups of proteins (such as the ε-amino groups of Lys). AGEs indirectly influence collagen formation and diminish NO bioavailability, thereby promoting collagen cross-linking [118]. Several drugs are currently commonly used to reduce AGE levels, including statins, also known as hydroxymethylglutaryl-coenzyme A reductase inhibitors, which are cholesterol-lowering drugs that affect AGEs. For example, simvastatin prevented AGEs-induced angiogenesis and disrupted related signaling pathways.

In addition, among angiotensin-converting enzyme inhibitors (ACE inhibitors), ramipril reduced the accumulation of AGE. Another new class of glucose-lowering drugs, sodium-glucose cotransporter 2 inhibitors, such as dapagliflozin, reduces AGE-mediated consequences of AGEs and RAGEs (receptor-mediated AGEs) and their downstream signaling interactions in diabetic rats [119].

### Therapies targeting FN

FN, a glycoprotein present in blood and tissues, plays a key role in matrix deposition by interacting with cellular and extracellular components [120]. There are several inhibitors of FN. For example, functional upstream domain (FUD), which is derived from the bacteria Adnexin Protein, has been identified as a strong FN matrix assembly inhibitor with a high binding affinity for FN’s 70 kDa N terminal region. It can reduce excessive ECM accumulation [121]. In addition, baicalein significantly reduced FN by inhibiting the expression of calpain-2 and vimentin, increasing the expression of E-cadherin, up-regulation of N-cadherin, vimentin, and Snail, and ERK signaling pathway [121]. Furthermore, experimental data from in vitro studies on hepatic stellate cells indicate that pUR4, which inhibits FN assembly, reduces the deposition of FN and collagen in the extracellular matrix, with no influence on TGF-β or TNF-α [122].

Notably, due to the importance of the reexpression of FN isoforms containing EDA or EDB domains in tumors, these isoforms are also referred to as carcinofetal forms and are significant markers of angiogenesis. Therefore, EDA and EDB domains have been widely utilized for the targeted delivery of therapeutic agents such as cytokines, cytotoxic drugs, chemotherapy drugs, and radioisotopes to tumors expressing FN, thus playing a significant therapeutic role in both primary cancer and metastatic lesions [123]. For example, CAR targets the FN EDA, which is highly expressed in the tumor stroma of a variety of tumors but not in healthy tissues. Using CAR-T cells targeting FN superdomain A for cancer therapy, EDA CAR-T therapy showed antiangiogenic effects and markedly reduced genetic signatures related to EMT, collagen synthesis, ECM organization, and IL- 6- STAT5 and KRAS pathways [116]. Similarly, EDB can also be used as a target for antibody-mediated radioisotope delivery, and fusion protein I labeling of L19 and small immune protein can not only slow tumor growth but also prolong the survival of F9 teratoma and head and neck cancer xenograft mice [8]. In addition to drug delivery, EDA and EDB-based vaccines appear prospective for treating and preventing certain types of cancer.

### Therapies targeting HA

HA is an important component of ECM. Increased HA can lead to decreased elasticity of tumor tissue, increased interstitial fluid pressure and vascular collapse, and inhibit drug delivery [37]. Current therapeutic strategies for HA are (i) hyaluronidase (HYAL) and chemotherapeutic drugs; (ii) the use of HA oligosaccharides (oHA) to induce apoptosis; (iii) HA inhibitors; and (iv) HA-based drugs.

Several preliminary reports have suggested that the addition of HYAL to chemotherapy regimens can significantly improve efficacy; for example, expression of the wild-type HYAL-1 variant triggers apoptosis in bladder cancer cells by inducing a G2-M phase arrest [124]. However, the animal HYAL used in these studies limits its therapeutic efficacy due to the immune response. The newly developed recombinant human HYAL, which can break down HA and be used to enhance drug delivery, has recently entered clinical trials; for example, enzymatic HA partially restores impaired perfusion of hepatic MCCs after anti-VEGF therapy and prolongs survival when combined with anti-VEGF therapy and chemotherapy [45]. In addition, oHA can induce apoptosis in lymphoma cell lines through inhibition of the downstream pathway of cancer survival. Physiologically, oHA inhibits the production of PIP3, participates in the activation of P13-K, and results in Akt inactivation (decreased by phosphorylation) without being involved in the activation of NF-κB [125].

Furthermore, it has been demonstrated that 4-methylumbelliferone (4-MU) and imatinib can decrease the accumulation of HA in the pancreas and prostatic carcinoma. Subsequently, the reduction of HA-mediated CD44 activation resulted in the downregulation of PI3K, Akt, and ERK signaling pathways, leading to impairment of the migration and invasion of the cells [70]. Mechanistically, MU consumed uridine diphosphate glucuronic acid, a precursor of ha, and reduced HAS mRNA expression; however, the inhibitory effect depended on the molecular weight of HA, because HMW-HA eliminated both inhibition and nonmetastasis of MU on MIA pCA-2 cells [1].

In addition, HA enhances the effect of anti-cancer agents by enhancing their solubility and biocompatibility. (i) HA-conjugated drug, (ii) HA-encapsulated drug, (iii) polymer, (iv) micelle, and (v) nanocarrier. For example, the drug named HYTAD1-p20 conjugated with HA-PTX can effectively inhibit cell mitosis [126]. Similarly, HA-conjugated PTX-N-hydroxy succinimidyl ester and HA-SN-38 (a CPT11 (irinotecan) metabolite) used in cell lines expressing CD44 (colon, breast, stomach, and others) reduced tumor cell growth and metastasis [81]. In addition, A novel self-assembling nanoparticle, consisting of a conjugate of HA and triphenyl phosphate, was able to degrade mutant p53 protein and synergistically deliver AMG510. Treatment of KRAS-TP53 co-mutations in gastrointestinal cancers by inhibition of mutant KRAS and mutant p53 signaling [127].

Although HA is considered a potential therapeutic target in cancer treatment, recent studies have shown that HYAL treatment may lead to a significant increase in glycolysis in tumor cells by degrading thioredoxin-interacting protein RNA, thereby upregulating glucose transporter 1 and increasing glucose uptake. It also promotes the migration and metastasis of tumor cells [21]. This suggests that there may be adverse consequences when using HYAL to break down HA, resulting in increased metastasis. Therefore, the potential negative effects need to be carefully considered when developing and applying therapies targeting HA.

### Therapies targeting CAF

CAFs are key components of the TME [128], which can enhance the ECM stiffness by the secretion of collagen and other fibrous macromolecules, as well as the degradation of this network through the release of proteolytic enzymes like MMPs, thereby improving the cellular mobility of the entire ECM [129]. Moreover, CAFs significantly contribute to tumor invasion, angiogenesis, progression, and metastasis by secreting multiple growth factors and proinflammatory cytokines, such as VEGF, IL-6, CXCL12, and TGF-β. Consequently, CAFs are recognized as significant targets in cancer therapy. Strategies for targeting CAF can be broadly divided into the following types: (1) Small molecule drugs targeting CAF; (2) Monoclonal antibodies targeting CAF; (3) Reprogramming CAF; (4) Combination therapy; and (5) Sequential delivery of small molecules and nanocarries.

Although CAFs play an important role in the TME, blind targeting of these cells can lead to adverse consequences. For example, in a transgenic mouse model of PDAC, researchers found that deleting α-SMA-expressing myofibroblasts led to more aggressive, undifferentiated tumors. These tumors showed enhanced hypoxia, EMT, and CSC features, while the survival of the animals was significantly reduced. A low number of myofibroblasts in the tumor is also associated with reduced survival in PDAC patients [130]. The results suggest that more careful and comprehensive consideration is needed when developing therapeutic strategies against CAFs.

### Therapies targeting TGF-β

TGF-β is a cytokine secreted by fibroblasts [129], which induces fibrosis by stimulating the secretion of ECM and its secretion through a positive feedback mechanism. It is deposited as a latent form in the ECM and requires integrin activation to obtain the active form [21]. TGF-β inhibitors not only normalize tumor stroma by reducing collagen I content but also improve the ability of other anticancer drugs to penetrate tumors more effectively [131], resulting in a significant reduction in tumor growth and metastasis. To date, preclinical and clinical data on TGF-β inhibitors, namely: (i) ligand traps; (ii) fusion proteins; (iii) monoclonal antibodies; (iv) antisense oligonucleotides; (v) small molecule receptor kinase inhibitors; and (vi) peptide aptamers.

## Targeting downstream signaling pathways of ECM stiffness

### Therapies targeting MMP

The dense ECM comprises minute pores that constitute a biophysical barrier hindering cell migration. The major group of enzymes responsible for the degradation of collagen and other proteins in the ECM is MMP [132]. MMPs are involved in tumor invasion, angiogenesis, and metastasis, thus rendering them ideal pharmacological targets for cancer therapy [133]. Various therapies, known as MMP inhibitors (MMPI) have been developed to target MMP in an attempt to control its enzymatic activity. Although in most cases, clinical trials of these compounds have not shown the expected results [134], current inhibitors targeting MMPs can be divided into two main categories: direct inhibitors and indirect inhibitors. Among the direct inhibitors are peptide-mimetic, non-peptide-mimetic MMPI, chemically modified tetracycline, and natural MMP inhibitors [135].

1. *Peptides-mimetic MMPIs*
  The peptide functions as a competing inhibitor by simulating the collagenous structure of the MMP cleavage site and chelating the zinc ions in the active site. Based on binding and chelating zinc ions, the peptide is subdivided into hydroxamic acid, carboxylate, hydrocarboxylate, sulfhydryl, and phosphate derivatives. The first MMP inhibitor to enter clinical trials was batimastat, a low molecular weight hydroxamic acid derivative with low water solubility that inhibits a variety of MMPs [135]. To overcome the solubility factor, another hydroxylate-based inhibitor, marimastat, was introduced for oral administration; however, marimastat showed musculoskeletal pain, possibly due to a wide range of inhibition [136].
2. *Non-peptide mimetic MMPIs*
  According to the currently known 3D conformation of the active site of MMP, non-peptoid MMPs were synthesized to improve specificity and oral bioavailability. This generation includes carboxylic acids such as BAY12-9566, prinomastat (AG3340), BMS-275291, CGS27023A, Ro-28-2653, S-3304, DX-2400 and cis-ACCP. BAY12-9566, a carboxylate, selectively inhibits gelatinase and stromelysin 1. Clinical trials of BAY12-9566 were stopped because of faster tumor growth in patients treated with the drug [137]. In addition, anti-tumor efficacy was observed with prinomastat either alone or in combination with chemotherapy in chemoresistant non-small cell lung cancer models. Unfortunately, prinomastat failed in Phase III trials owing to its ineffectiveness in patients with advanced disease [21].
3. *Chemically modified tetracyclines (CMTs)*
  Chemically modified tetracyclines (CMTs) lack antibiotic activity and inhibit MMP enzymatic activity and synthesis by impeding gene transcription. This family includes SB-3CT, temocycline, CMT-3 (Col3), minocycline, and doxycycline. SB-3CT, in particular, inhibits gelatinase through the enzymatic ring opening of thioxane, yielding stable zinc thiosulfide [133]. It has been reported to inhibit liver metastasis and improve survival in mouse models [138].
4. *Natural MMP inhibitors*
  TIMP is a natural inhibitor of MMPs [137], but due to its low bioavailability, potential side effects, and toxicity, its clinical application is limited and it has not been widely used to inhibit MMPs in clinical practice [139]. To prevent adverse effects and toxicity from synthetic MMPI, the field of natural compounds provides an answer. Consider neovastat compound extracted from shark cartilage, which inhibits not only MMP enzyme activity but also VEGF [140]. Another natural drug with anticancer effects is genistein, a soy isoflavone compound structurally similar to estradiol. In addition to estrogenic and antiestrogenic properties, genistein also imbues tumor growth inhibition and invasion, but due to its poor water solubility, short half-life, and complex multi-target mechanism of action, it has not been widely used in clinical practice to inhibit MMPs.
5. *Indirect MMP inhibitors*
  Indirect MMP inhibitors include blockade of signal transduction pathways (such as MAPK or ERK pathways [141]), and gene therapy (such as inhibition of miRNA expression [51]) to achieve the inhibition of MMP [142].
  However, in most cases, clinical trials of these compounds have not shown the expected results, mainly for the following reasons:
  **Selectivity issues of broad-spectrum MMPIs**: Broad-spectrum MMPIs may inhibit MMPs with antitumor effects, leading to tumor progression.
  **Clinical trial stage selection**: Most clinical trials of MMPIs have been conducted in patients with advanced tumors, and the role of MMPs is more critical in the early stages of tumors. Therefore, MMP-targeted therapy should be designed to test its anti-metastatic properties in early-stage tumors, and more selective MMP inhibitors should be developed and used without affecting other protective MMPs [21].

### Therapies targeting integrins

The term “integrin” was originally used to describe the function of the receptor to integrate the ECM network with the cytoskeletal network. It is a member of the membrane glycoprotein superfamily [21]. Integrins are transmembrane (TM) cell surface heterodimeric receptors composed of an α subunit and a β subunit. In mammals, integrins have 18 α subunits and 8 β subunits, forming 24 αβ integrin heterodimers. Integrins process biochemical and mechanical signals between cells and their environment in a variety of health and disease states [90].

TGF-β1 is a key factor in the development of fibrosis and is considered a potential therapeutic target. TGF-β1 can effectively prevent fibrosis without causing severe multiple organ dysfunction However, clinical trials have shown that directly targeting TGF-β1 to treat fibrotic diseases is not feasible. Instead, studies have found that blocking the interaction between integrins, particularly those rich in αv subunits, maybe a more effective approach [143]. Recent evidence suggests that αvβ3, α4β7, α9β1, and α8β1 integrins can inhibit the progression of nonalcoholic steatohepatitis to liver fibrosis [144]. In addition, α3β1, α4β1, αvβ8, and α8β1 integrins play a role in pulmonary fibrosis. These studies showed that integrin inhibitors are promising in treating fibrotic diseases [145].

Moreover, targeting integrins can not only reduce fibrosis but also achieve antitumor effects through a variety of mechanisms. For example, they can reduce PD-L1 expression in cancer cells, prevent T-cell homing to the tumor, and inhibit angiogenesis, thereby reducing tumor growth and metastasis. These findings further highlight the potential value of integrin inhibitors in cancer therapy. However, targeting integrin therapy has been a challenge due to the large number and complexity of integrins and the overlapping downstream pathways regulated by integrin heterodimers [21]. Thus far, only seven integrin-targeting drugs, including abciximab, eptifibatide, tirofiban, natalizumab, vedolizumab, lifitegrast, and carotegrast, have been successfully marketed. In the future, besides focusing on the effectiveness of integrin antagonists, we should also be concerned about the adverse reactions of integrin antagonists in clinical applications or clinical trials, and develop more effective drugs to meet the clinical needs.

### Therapies targeting FAK

FAK is a cytoplasmic protein tyrosine kinase [143]. Changes in matrix composition or stiffness, cytokines, growth factors, integrins, and pH trigger FAK activity through the αvβ5/FAK/PI3K/Akt signaling pathway [146] to prevent anoikis and other types of cell death. FAK promotes tumor invasion, metastasis, drug resistance, and self-renewal of CSCs [147]. Small-molecule FAK inhibitors have been shown to inhibit the growth and metastasis of cancer in a number of preclinical models. Compounds that target FAK can be divided into ATP-competitive kinase inhibitors (KI), molecules that block FAK catalytic activity by alternative means (aKI), and compounds that target FAK scaffolds (scaffold inhibitors, SI) [147]. Defactinib (VS-6063) is a potent, ATP-competitive, and reversible dual inhibitor of FAK and PYK2, and Defactinib is being evaluated in multiple clinical trials for its effect in combination therapy (NCT02546531, NCT00787033) [148]. Defactinib combined with PTX treatment can significantly reduce cell proliferation and increase apoptosis, resulting in tumor weight loss (clinical trial number: NCT02546531) [21]. Mechanically, Defactinib therapy decreased Akt and YB-1 levels and increased chemosensitivity in taxane-resistance cells.

The safety profile of defactinib was acceptable and the adverse reactions associated with treatment were mild to moderate and reversible, making it a promising candidate. 1H-Pyrrolo (2, 3-B) pyridine acts as an allosteric FAK kinase inhibitor through induction of DFG-loop conformation, binding to FAK hinge region and inhibiting kinase activity [28]. Chloropyramine hydrochloride is a compound that targets the FAK scaffold and blocks the protein–protein interaction between FAK and VEGFR-3. Chloropyramine hydrochloride inhibits the proliferation and growth of cancer cells by inhibiting VEGFR-3 and FAK signaling [149].

### Therapies targeting YAP/TAZ

YAP and TAZ can be regarded as a “link” by which tumor cells reprogram their surrounding ecosystem into a resilient, stiff, growth-promoting, and immune cold TME [5]. Their activation positively correlates with malignancy, recurrence, metastasis, reduced overall survival, and drug resistance [150].

Virteporfin is the first drug capable of inhibiting both the binding of YAP/TAZ to TEAD and YAP/TAZ-induced transcription and activity. Currently, it is primarily used for maculopathy, but its potential as an anti-tumor agent is being investigated [151]. Moreover, currently, most drugs in YAP/TAZ clinical trials target the combination of YAP/TAZ and TEAD, and all are in Phase I or II in solid tumor clinical trials [152]. For example, K-975, a selective TEAD inhibitor, binds to cysteine residues within the TEAD palmitic acid-binding pocket, thereby inhibiting the YAP/TAZ-TEAD interaction. Another drug targeting the hydrophobic pocket of TEAD is VT3989 developed by Vivace Therapeutics, which has entered Phase I clinical trials (NCT04665206) for the treatment of NF2 mutated solid tumors and mesothelioma [153].

CPD3.1, TED-347, MGH-CP1, IK-930, and other drugs can block the YAP-TAZ-TEAD interaction by targeting different pockets of TEAD. Notably, YAP and TAZ are transcriptional coactivators not only of TEAD but also of AP-1, CKB [154] and STAT3 [18]. In addition, YAP/TAZ activity can be affected by inhibiting the YAP signaling pathway, such as Src family kinase inhibitors (e.g., dasatinib, salatinib, bosutinib, and imatinib) [21], small molecule agonists of GPCR signaling (e.g., LPA or S1P agonists), and other mechanisms. Inhibitors of the Wnt/β-catenin pathway (such as the tankyrase inhibitor XAV939) [155], inhibitors of Rho-GTPases and ROCK (such as fasudil) [156], and modulators of the SREBP/mevalonate pathway (e.g., statins or bisphosphonates) can all attenuate YAP/TAZ activity [157]. In addition, utilizing the CRISPR/Cas9 gene editing system to knock down YAP expression can hinder the proliferation of diffuse large B-cell lymphoma cells and trigger cell cycle arrest [65] (Tables 1 and 2).

**Table 1:** Therapies targeting CAF

Therapy	Drug	Mechanism	
Small Molecule Drugs	PT-100	Small molecule compounds inhibit specific signaling pathways (such as FAP, TGF-β, Hh, etc.) to diminish CAF activation and proliferation	Dipeptidyl peptidase inhibitor targeting FAP, reduces CAF accumulation, and improves chemotherapy response rate [158]
	Val-boroPro (Talabostat)		FAP inhibitor, used in Phase II clinical trials in metastatic colorectal cancer, but with unsatisfactory efficacy, probably due to incomplete inhibition of targeted enzymes in CAF [159]
	Metansine (DM1)		Anti-FAP antibody binding cytotoxic drug that has shown promising results in experimental models [160]
	Sonidegib		Hh signaling pathway inhibitor, combined with docetaxel in patients with advanced TNBC, has shown some antitumor activity [161]
	Pirfenidone		Targeting TGF-β pathway activation in CAFs has been demonstrated to enhance the effect of chemotherapy and immunotherapy in breast cancer and pancreatic tumor models [70]
	Vismodegib		This targets Hedgehog in CAFs and is FDA-approved for the treatment of basal cell carcinoma. However, more research is needed to treat other CAF-dense solid tumors, such as breast and pancreas carcinoma
	Repurposed drugs (Tranilast, etc.)		Including antihistamines (tranilast, ketotifen), antihypertensives (losartan, bosentan), and corticosteroids (dexamethasone)[162]
Monoclonal Antibodies	Mab F19 (Sibrotuzumab)	These bind with high affinity to surface markers like FAP, reducing CAF accumulation and augmenting chemotherapy effects	Human anti-FAP antibody,, was used in a Phase I clinical trial and showed safety and modest efficacy, but did not meet the requirements for a partial or complete response [163]
	MAb FAP5-DM1		A novel antigen-drug conjugate, targeting FAP, has shown excellent therapeutic efficacy and durable tumor suppression in xenograft mouse models [164]
	RO6874281		FAP-targeting antibody, which binds to FAP with high affinity, is currently undergoing Phase I and II clinical trials to evaluate its effect in combination with chemotherapy drugs or PD-L1 antibody [163]
Reprogramming CAF	Calcitriol	The CAF can be reprogrammed to a quiescent state by specific molecules (such as vitamin D-like ligand, lipogenin A4, etc.) or silencing specific genes (eg, ITGA11 and ITGA5) to reduce its ability to promote tumor growth [165]	A vitamin D-like ligand that restores activated pancreatic stellate cells to quiescence by RA treatment, reducing inflammation and fibrosis markers, and enhancing gemcitabine efficacy of the chemotherapeutic drug GEM
	Lipoxin A4		An endogenous bioactive lipid that inhibits tumor growth by targeting FPR2 to reduce CAF activation and migration
	Au@PP/RA/SiHSP47		Gold nanoparticles that enhance chemotherapy by maintaining PSC quiescence and reducing collagen content through vitamin A, RA, and SiHSP47
	sTRAIL		An engineered TNF-related apoptosis-inducing ligand delivered via lipid-coated protamine DNA complexes, inducing CAF and tumor cell apoptosis [163]
	Knockdown of satellite transcripts		Attenuating cellular senescence and preventing inflammatory CAF phenotypes, thereby inhibiting pro-tumor functions [166]
Combination Therapy	DGL/GEM@PP/GA	The therapeutic effect can be improved by combining multiple drugs or nanocapsules to inhibit the function of CAF and tumor cells at the same time	Multifunctional size-switching nanoparticles that co-deliver GEM and GA, down-regulate the DRP molecule Wnt16, and significantly inhibit the growth of pancreatic cancer and breast cancer [167]
	LCP-QP NP		Lipid/calcium/phosphate nanoparticles coated with quercetin prodrug, combined with LPC NP, significantly enhancing matrix-rich UMUC3 bladder tumor inhibition and decreasing Wnt16 expression [168]
	TSL/HSA-PE		Thermosensitive liposomes co-delivering PTX and EA, releasing drugs through local heating, significantly reducing CAF activity and inhibiting tumor growth [163]
	muFAP-CAR T cells		Enhancing endogenous CD8 + T cell antitumor responses to safely and effectively inhibit tumor growth [169]
	Oral FAP DNA vaccine		A DNA vaccine that targets FAP, which is highly expressed in the tumor stroma, to specifically kill CAFs, reduce collagen I expression, and increase the uptake of chemotherapy drugs, has successfully inhibited the growth and metastasis of multidrug-resistant tumors [170]
	MEKi/STAT3i/PD-1 blockade		Co-treatment with MEKi and STAT3i reduced pro-inflammatory CAF and myofibroblast-like CAF phenotypes, enriched Ly6a/Cd34 expressing CAF, which exhibited mesenchymal stem cell-like features and promoted the reprogramming of tumor-associated macrophages and CD8 + T cell homing. Significantly improved anti-tumor response and survival in PKT mice, and clinically achieved positive effects in patients with chemotherapy-refractory pancreatic ductal adenocarcinoma (PDAC) [171]
Sequential delivery of small molecules and nanocarriers	Tranilast	stepwise delivery of small molecules and nanocarriers to progressively reduce CAF activity and improve the tumor penetration and therapeutic efficacy of nanocarriers	Antifibrotic agent administered first to down-regulate CAF activity followed by administration of docetaxel micelles to produce a synergistic antitumor effect [172]
	Frax-NPCGKRK + SiKras-LCPApoE3		Nanoparticles loaded with antifibrotic drugs were first administered to reduce CAF activity, followed by nanoparticles loaded with siRNA to silence oncogenic KRAS mutations and effectively kill cancer cells [173]
	Losartan		Angiotensin II antagonist, which improves the TME by pre-injection of losartan-encapsulated liposomes, and enhances the accumulation and penetration of subsequent delivery nanocapsules in the tumor [113]

**Table 2:** Therapies targeting TGF-β

Therapy	Drug	Mechanism
Ligand Traps	AVID200	An engineered TGF-β ligand trap for the treatment of solid tumors and myelofibrosis
Fusion Proteins	Bintrafuspα	A pioneering dual-function fusion protein that targets both PD-L1 and TGF-β [174, 175] has undergone evaluation in several Phase 2 trials for biliary tract cancer, non-small cell lung cancer, cervical cancer, and other solid tumors. Findings suggested encouraging clinical efficacy in advanced solid tumors [176], and did not report any dose-limiting toxicity [177]
	SHR-1701	Developed by Henrui Pharma [165], Studies demonstrated no dose-limiting toxicity, and the maximum tolerated dose was not reached [178]
	Tβrii-fc	A recombinant Fc fusion protein incorporating the soluble ectodomain of TβRII has been shown to reduce the incidence of mammary tumor metastasis in transgenic mice [179]
	sTβRII-Fc	When coupled with an oncolytic adenovirus, it inhibits TGF-β signaling and reduces tumor growth in vitro and nude mouse models [110]
Monoclonal Antibodies	1D11	A pan-neutralizing antibody against mouse TGF-β binding to three TGF-β isoforms, reducing their biological activity and inhibiting metastasis, as well as rescuing bone loss in animal models of lung and bone metastasis of breast cancer
	NI-5793	A fully human monoclonal antibody targeting TGF-β, specifically an IgG2 isotype, is being developed for the treatment of pancreatic cancer and myelofibrosis
	Dalutrafusp	A bifunctional monoclonal antibody designed to target both CD73 and TGF-β, two key molecules involved in immunosuppressive pathways [180]
Antisense Oligonucleotides	AP12009 (Trabedersen)	A phosphorothioate antisense oligonucleotide specifically targeting TGF-β2, which inhibits TGF-β2 expression and reduces the proliferation and migration of glioma cells in vitro
	STP705	A siRNA gene-silencing oligonucleotide that selectively inhibits TGF-β1 and COX-2, used for the treatment of conditions such as basal cell carcinoma
	TAS-001	An antisense oligonucleotide specifically targeting TGF-β2 is employed in the treatment of advanced or metastatic solid tumors [181]
Small-molecule receptor kinase inhibitors	Galunisertib	An oral small-molecule TGF-βRI tyrosine kinase inhibitor that down-regulates SMAD2 phosphorylation, thereby blocking TGF-β signaling, and has entered clinical trials [17]
	Sorafenib and regorafenib	To date, the only approved first-line and second-line treatments, respectively
	TGF-βRI tyrosine kinase inhibitor SB-431542	A TβRI inhibitor developed by GlaxoSmithKline that blocks TGF-β-induced FN and collagen transcription in vitro [182]
	LY364947	A pyrazole-based compound that inhibits the serine-threonine kinase activity of TGF-βR1. This study aimed to find compounds that could relieve the growth inhibitory effect of TGF-β1 on the mink lung Mv1lu cell line
	Ki26894	A TβRI/ALK5 kinase inhibitor, that blocks TGF-β signaling, invasion, and mobility in vitro and in vivo of bone metastatic breast cancer cells
	SD-208	In a mouse model of human melanoma, pretreatment of tumor cells prevented the onset of osteolytic bone metastases and significantly reduced the size of bone metastases during long-term treatment
	LY2109761	A dual inhibitor of TβRI/II, demonstrating the inhibition of metastatic development in a variety of short-term rat tumor models but developing tumor biochemical resistance and further cancer progression with long-term administration
	Ki26894	An ATBR-1 kinase inhibitor that reduces the invasiveness and EMT of sclerotic gastric cancer cells [109]
		Small molecules that mimic the structure and function of a specific protein or peptide, often used to target specific signaling pathways
Peptide Aptamers	Trx-SARA	A peptide aptamer that specifically binds Smad2 and Smad3 reduces the level of Smad2/3 in the TGF-β-mediated Smad4 complex and inhibits TGF-β-induced EMT in NMuMG mouse mammary epithelial cells in vitro [110]
Other Anti-Fibrotic Drugs	Ajulemic Acid	A synthetic cannabinoid derivative, which has been shown to have anti-fibrotic and anti-inflammatory effects. The mechanism of action is through the activation of cannabinoid receptor 2, which produces prostaglandins, reduces inflammatory cytokines, and inhibits TGF-β production [21]

### Therapies targeting DDR

Discoidin domain receptor (DDR) belongs to the family of receptor tyrosine kinases as a non-integrin collagen receptor [183]. The molecular structure of DDR comprises three main parts: the extracellular binding domain, the transmembrane TM domain, and the intracellular kinase domain (KD) [184]. The extracellular binding domain consists of a discoidin domain and a discoidin-like domain responsible for collagen binding. The TM domain includes an extracellular juxtamembrane region, which contains phosphorylated tyrosines that act as DDR-binding proteins, and a TM helix that facilitates the dimerization of collagen-independent receptors. The intracellular domain includes an intracellular juxtamembrane region and a catalytic tyrosine KD that determines the enzyme’s intrinsic activity [185]. Upon interaction of the collagen-bound discoin domain with collagen, The conformation of DDRs is altered, followed by phosphorylation of KD, which results in the binding of the adapter proteins, including ShcA and Nck2 to the cytoplasmic domain of DDRs. Both integrins and DDRs sense the stiffness of the ECM and transduce this signal into the cell [157]. DDR exists in two isoforms: DDR1 and DDR2 [128, 186–189]. DDR1 is broadly expressed in epithelial cells, whereas DDR2 is predominantly found in mesenchymal cells [185]. Notably, only DDR2 Activation of Fibrous Collagen, specifically types I and III [190].

Emerging studies have demonstrated that DDR1 is a key regulator of cancer genesis, differentiation, migration, and invasion [191]. Aguilera et al. demonstrated that pharmacological inhibition of DDR1 using an ATP-competitive, orally bioavailable small-molecule kinase inhibitor reduced colony formation and migration of pancreatic tumor cells [192]. Inhibition of DDR1 kinase activity has also been reported to diminish the invasive and metastatic potential of patient-derived circulating colorectal cancer cell lines. Owing to the structural similarity of the KD, several putative tyrosine kinase inhibitors [157], such as dasatinib, salatinib, bosutinib, imatinib, and nilotinib [193], effectively block the kinase activity of either DDR1 or DDR2 [194]. In addition, a number of new selective DDR inhibitors have been developed. For instance, DDR1-IN-1 and DDR1-IN-2 can inhibit DDR1 phosphorylation effectively (Fig. 5b) [195].

## Discussion

There is increasing evidence that cancer development is strongly influenced by the physical properties of the extracellular matrix, particularly its stiffness. When a tumor forms, the ECM becomes stiffer. This trend can be attributed to a variety of factors, including enhanced matrix deposition, collagen cross-linking, fiber hardening, elevated cell density, elevated interstitial fluid pressure, and heightened cell-to-cell interactions. Sclerotic ECM is not only a common feature of various types of cancer, but also an important factor in cancer progression because sclerotic ECM enhances cancer cell growth, survival, and migration, thereby promoting EMT. In addition, ECM stiffness constitutes a vicious cycle that further drives cancer progression. Therefore, targeting the receptors and signaling pathways that induce ECM stiffness may be an effective means to reduce matrix stiffness. Key mediators such as integrins, TGF-β, and YAP/TAZ play crucial roles in inducing ECM sclerosis, while multiple signaling pathways (such as Notch, Wnt, Rho) are also involved. The interaction between these signaling pathways has not been fully elucidated. Results in unintended physiological consequences and increased treatment complexity. Hence, there is an urgent need to develop advanced diagnostic and imaging tools for precise tumor stiffness assessment, which is crucial for evaluating patient prognosis and immunotherapy response. For example, employing nano-delivery systems for the simultaneous administration of all-trans retinoic acid and siRNA targeting HSP47 can inhibit tumor cell proliferation and induce pancreatic stellate cells into a quiescent state, thereby resolving pancreatic fibrosis [196]. Moreover, normalizing the stroma, targeting signaling pathways induced in stromal cells, or cytokines secreted by tumors, rather than completely ablating the stroma, may be a more effective strategy to improve outcomes (Table 3).

**Table 3:** Clinical trials of drugs targeting ECM stiffness or downstream signals (clinicaltrials.gov)

Target	Drugs	Conditions	NCT number	Phase	Status
TGF-β	Bintrafusp alfa	Uterine Cervical Neoplasms	NCT04246489	II	Recruiting
	Galunisertib	Metastatic Pancreatic Cancer	NCT02734160	I	Completed
	TEW-7197	Advanced Stage Solid Tumors	NCT02160106	I	Completed
	LY2157299	Hepatocellular Carcinoma	NCT02178358	II	Completed
	Halofuginone	Unspecified Adult Solid Tumor, Protocol Specific	NCT00027677	I	Completed
	Fresolimumab	Stage IA Non-Small Cell Lung Carcinoma Stage IB Non-Small Cell Lung Carcinoma	NCT02581787	I/II	Completed
	Losartan	Glioblastoma Brain Metastases	NCT03951142	II	Enrolling by invitation
	FOLFIRNINOX	Pancreatic Adenocarcinoma Pancreatic Adenocarcinoma Metastatic	NCT05077800	II	Recruiting
	S-3304	Solid Tumors	NCT00033215	I	Completed
Collagan	Collagenase	Dupuytren’s Contracture	NCT00004409	II	Completed
	D-Penicillamine	Head and Neck Cancer Recurrent Cancer	NCT06103617	II	Recruiting
LOX	ATN-224	Breast Cancer	NCT00674557	II	Terminated
	PXS-5505	Myelofibrosis	NCT04676529	I/II	Active, not recruiting
LOX	PXS-5382A	Healthy adults	NCT04183517	I	Completed
LOXL2	Simtuzumab (GS-6624)	Liver Fibrosis Due to NASH	NCT01672879	II	Terminated
	Epigallocatechin-3-gallate (EGCG)	Idiopathic Pulmonary Fibrosis	NCT03928847	I	Completed
	PXS-5505	Myelofibrosis	NCT04676529	I/II	Active, not recruiting
	GB2064	Myelofibrosis	NCT04679870	II	Active, not recruiting
LOXL2	PAT-1251	Myelofibrosis Transformation in Essential Thrombocythemia Polycythemia Vera, Post-Polycythemic Myelofibrosis Phase Primary Myelofibrosis	NCT04054245	II	Withdrawn
AGE	Ramipril	Atherosclerosis	NCT01053910	IV	Completed
	Dapagliflozin	Azheimer disease	NCT03801642	I/II	Completed
CAF	Sonidegib	Locally Advanced Basal Cell Carcinoma	NCT04806646	II	Recruiting
	Vismodegib	Basal Cell Carcinoma	NCT02639117	I	Completed
	Sibrotuzumab	Carcinoma, Non-Small-Cell Lung	NCT02209727	I	Terminated
	RO6874281	Solid Tumor Breast Cancer Cancer of Head and Neck	NCT02627274	I	Completed
	68 Ga-FAPi-46	Cancer of Unknown Primary Site	NCT05263700	Not Applicable	Recruiting
	68 Ga-FAP-2286	Solid Tumor	NCT04939610	I/II	Recruiting
	PET/CT ([F-18] FAPI-74)	Neoplasms	NCT05442151	Not Applicable	Recruiting
MMP	18-FDG	Solid Tumor	NCT06719856		Not yet recruiting
	Doxycycline	Lymphangioleiomyomatosis Tuberous Sclerosis	NCT00989742	IV	Completed
	COL-3	Lymphoma Melanoma Neoplasm Metastasis 1 more	NCT00001683	I	Completed
	Prinomastat	Brain and Central Nervous System Tumors	NCT00004200	II	Completed
	S-3304	Solid Tumors	NCT00033215	I	Completed
	Marimastat	Lung Cancer	NCT00003011	III	Completed
	Prinomastat (AG3340)	Lung Cancer	NCT00004199	III	Completed
	Neovastat (AE-941)	Kidney Cancer	NCT00005995	III	Completed
	Genistein	Colon Cancer Rectal Cancer Colorectal Cancer	NCT01985763	I/II	Completed
	COL-3	Lymphoma Melanoma Neoplasm Metastasis 1 more	NCT00001683	I	Completed
	Tobramycin	Pancreatic Cancer	NCT01693523	II	Completed
Integrin	Abciximab	Acute Myocardial Infarction	NCT00894023	III	Terminated
	Cilengitide	Glioblastoma	NCT00689221	III	Completed
	ATN-161	Malignant glioma	NCT04177108	I/II	Completed
	MEDI-522	Metastatic mela-noma	NCT00066196	II	Completed
	Eptifibatide	Acute Coronary Syndrome	NCT01919723	II	Completed
	Tirofiban	Acute Myocardial Infarction	NCT00300833	IV	Unknown status
	Natalizumab	Primary Progressive Multiple Sclerosis Secondary Progressive Multiple Sclerosis	NCT01077466	II	Completed
	Vedolizumab	Crohn’s Disease	NCT02674308	IV	Terminated
	Lifitegrast	Dry Eye Disease	NCT02284516	III	Completed
FAK	Defactinib (VS-6063)	Non Hematologic Cancers	NCT01943292	I	Completed
	VS-4718	Non Hematologic Cancers Metastatic Cancer	NCT01849744	I	Terminated
	GSK2256098	Cancer Neoplasms	NCT01938443	I	Completed
	SIGX1094R	Advanced Solid Tumors	NCT06739291	I	Recruiting
	VS-6766	NSCLC Low Grade Serous Ovarian Cancer Endometrioid Carcinoma	NCT03875820	I	Active, not recruiting
	PF-04554878	Cancer	NCT00787033	I	Completed
	CT-707	Advanced Pancreatic Cancer	NCT05580445	I/II	Recruiting
	PF00562271	Head and Neck Neoplasm Prostatic Neoplasm Pancreatic Neoplasm	NCT00666926	I	Completed
YAP/TAZ	Verteporfin (VP)	Solid Tumors, Adult Solid Tumor Malignant Pleural Mesothelioma (MPM) 5 more	NCT06381154	I	Terminated
	IK-930	Solid Tumors, Adult Solid Tumor Malignant Pleural Mesothelioma (MPM) 5 more	NCT05228015	I	Terminated
	VT3989	Solid Tumor, Adult Mesothelioma	NCT04665206	I/II	Recruiting
SRC	Dasatinib	Malignant Solid Tumour	NCT00388427	I	Completed
β-catenin	PRI-724	Advanced Pancreatic Cancer Metastatic Pancreatic Cancer Pancreatic Adenocarcinoma	NCT01764477	I	Completed
ROCK	Fasudil	Cognitive Decline, Mild Alzheimer Disease	NCT06362707	II	Recruiting
CD33	CRISPR/Cas9 Gene Editing	Relapsed/Refractory Acute Myeloid Leukemia (AML)	NCT05662904	I	Not yet recruiting
DDR1	DAT-2645 tablet	Solid Cancers BRCA Mutation HRD Cancer	NCT06614751	I	Not yet recruiting
	Elimusertib (BAY1895344)	Advanced Solid Tumors	NCT04095273	I	Completed

Additionally, reducing ECM stiffness may accelerate invasion and metastasis by weakening the mechanical barrier effect on tumor cells. For instance, in breast cancer, soft matrices and low cytoskeleton tension may facilitate the development of EMT in breast cancer cells. Very low mammogram density (< 10%) indicates poor prognosis and histological grade of tumor [52]. Therefore, when targeting the ECM, it is crucial to design personalized treatment regimens. Furthermore, an accurate assessment of tumor stiffness is essential for evaluating patient prognosis and guiding ECM stiffness therapy. Traditional methods for assessing tumor stiffness include ultrasound elastography, magnetic resonance elastography, shear wave elastography, and transient elastography. Nevertheless, these approaches frequently depend significantly on radionuclides and single fluorescence techniques and are constrained by potential adverse reactions and the challenge of imaging agents effectively penetrating dense solid tumors. Thus, there is an urgent need to develop diagnostic and imaging tools that can accurately assess tumor stiffness, which are essential factors in assessing patient outcomes and determining the efficacy of immunotherapy.

Thirdly, there is a lack of materials that can accurately mimic the ECM in vitro. To date, commonly used materials include collagen, gelatin, polyethylene glycol (PEG), Matrigel, gelatin, hyaluronic acid, chitosan, and alginate [197]. Among them, Matrigel from Engelberth-Holm-Swarm (EHS) mouse sarcoma contains a variety of ECM components, such as collagen, laminin and FN, which cannot accurately simulate the interaction and dynamics between different ECM components and is not very similar to real human tumors. Similarly, some natural ECM-derived hydrogels (such as collagen hydrogels) are expensive, lack reproducibility, and require extensive handling and specific equipment [198]. Therefore, with the continuous development of the field of biomaterials, new engineered materials with independently adjustable BM-like bioactivity and mechanical properties have been developed [199], including Synthetic hydrogels, Decellularized ECM, Microfluidic cell culture, and 3D bioprinting [200]. These materials have significantly improved in terms of composition controllability, dynamic response, and spatial heterogeneity, and have eliminated the first generation of natural source hydrogels. Among them, synthetic hydrogels can simulate the ECM of the human body, while avoiding the problems of heterogenicity and batch differences of natural materials [201]. Similarly, Decellularized ECM ideally removes all potential immunogenic components and preserves the original composition and structure of the natural ECM [202]. However, it is important to note that ineffective decellularization is often associated with a strong inflammatory response. Despite all the advances in the field [202], the therapeutic application of decellularized ECM still faces challenges in terms of standardization and scaling, as well as ethical and regulatory constraints. In addition, microfluidic cell culture is a method to culture cells in a micro-scale space using microfluidic technology. It mimics the ECM microenvironment in the body by precisely manipulating trace fluids through microfluidic devices, thus closer to the behavior of cells in the real physiological environment [203, 186–188]. Finally, 3D bioprinting is a promising innovative biomanufacturing strategy for precisely locating biologics [204], including living cells and ECM components [189], in prescribed 3D hierarchical tissues to create artificial multicellular tissues/organs [205].

In summary, tissue fibrosis and ECM stiffness are associated with the progression of many tumors, including hepatocellular carcinoma, pancreatic ductal adenocarcinoma, and breast cancer, among others. Although the mechanisms by which ECM regulates intracellular signaling have been extensively studied, how sclerotic ECM affects the tumor microenvironment and intercellular communication that promotes tumor growth is currently unknown. At present, it has been shown that the effect of ECM stiffness on tumor progression is not only related to intracellular signaling but also involves extracellular vesicles such as exosomes [206]. The increase of ECM stiffness will increase the secretion of exosomes, which will lead to changes in the TME, thereby promoting tumor growth. With the interdisciplinary integration of cell biology, oncology, materials science, nanotechnology, bioengineering, and other disciplines, the development of more accurate and efficient ECM simulation materials and treatment strategies, the formulation of individualized treatment plans, the overcoming of the complexity of multiple signaling pathways, and the optimization of drug delivery systems will not only help to improve the effect of cancer treatment and patient survival rate. It is also applicable to the research of other diseases, such as neurodegenerative diseases and cardiovascular diseases.

## Data Availability

No datasets were generated or analysed during the current study.

## Abbreviations

TME: Tumor microenvironment
ECM: Extracellular matrix
EMT: Epithelial-mesenchymal transition
CAFs: Cancer-associated fibroblasts
MSCs: Mesenchymal stromal cells
ECs: Endothelial cells
FN1: Fibronectin 1
TNC: Tenasin C
HA: Hyaluronic acid
HCC: Liver cancer
TGF-β: Transforming Growth Factor β
IL: Interleukin
ROCK: Rho-associated protein kinase
MMP: Matrix metalloproteinase
TIMP-1: Metalloproteinase inhibitor 1
HIF-1α: Hypoxia-inducible factor
PDGFRα: Platelet-derived growth factor receptor α
TNF-α: Tumor necrosis factor α
HSP47: Heat shock protein 47
HLCC: Hyl aldehyde-derived collagen acea
LCC: Lys aldehyde-derived collagen cross-linking
Hyl: Hydroxylysine
Lys: Lysine
FAK: Focal adhesion kinase
LOXL: LOX-like
IPN: Interpenetrating network
ERK: Extracellular signal-regulated kinase
PI3K: Phosphatidylinositol 3 kinase
BMs: Basement membranes
Akt: Protein kinase B
STAT3: Transcription 3
OPN: Osteopontin
BMDCs: Bone marrow-derived cells
CSCs: Cancer stem cells
PTX: Paclitaxel
MRP1: Multidrug resistance protein 1
5-Fu: 5-Fluorouracil
ILK: Integrin-linked kinase
ERα: Estrogen receptor-α
RGD: Arg-Gly-Asp
LMW: Low-molecular-weight
VEGFR-2: VEGF receptor 2
FGF-2: Fibroblast growth factor 2
APCs: Antigen presenting cells
DCs: Dendritic cells
LDHA: Lactate dehydrogenase A
MDSCs: Myeloid-derived suppressor cells
MET: Mesenchymal-epithelial transition
SOX4: SRY-Box transcription factor 4
TRPV4: Transient receptor potential vanilloid 4
ALP: Alkaline phosphatase
PFK: Phosphofructokinase
LDH: Lactate dehydrogenase
PGM1: Phosphoglucomutase 1
CKB: Creatine kinase type b
MUFA: Monounsaturated fatty acid
SFA: Saturated fatty acid
SCD1: Stearoyl-coa desaturase 1
DSB: Double-strand break
FUD: Functional upstream domain
HYAL: Hyaluronidase
oHA: HA oligosaccharides
4-MU: 4-Methylumbelliferone
PDAC: Pancreatic ductal adenocarcinoma
MMPI: MMP inhibitors
CMTs: Chemically modified tetracyclines
TM: Transmembrane
DDR: Discoidin domain receptor
KD: Kinase domain
